# Exploring the Therapeutic Mechanisms of Huzhang–Shanzha Herb Pair against Coronary Heart Disease by Network Pharmacology and Molecular Docking

**DOI:** 10.1155/2021/5569666

**Published:** 2021-11-30

**Authors:** Dan Li, Longtao Liu, Shengjie Yang, Yanwei Xing, Limin Pan, Ran Zhao, Yixi Zhao, Guirui Huang, Min Wu

**Affiliations:** ^1^Guang An'men Hospital, China Academy of Chinese Medical Sciences, Beijing 100053, China; ^2^Xiyuan Hospital, China Academy of Chinese Medical Sciences, Beijing 100091, China; ^3^The Third Affiliated Hospital, Beijing University of Chinese Medicine, Beijing 100029, China

## Abstract

**Background:**

Coronary heart disease (CHD) seriously affects human health, and its pathogenesis is closely related to atherosclerosis. The Huzhang (the root of *Polygonum cuspidatum*)–Shanzha (the fruit of *Crataegus* sp.), a classic herb pair, has been widely used for the treatment of CHD. In recent years, Huzhang–Shanzha herb pair (HSHP) was found to have a wide range of effects in CHD; however, its therapeutic specific mechanisms remain to be further explored. The aim of this study was to elucidate the molecular mechanism of HSHP in the treatment of CHD using a network pharmacology analysis approach.

**Methods:**

The Batman-TCM database was used to explore bioactive compounds and corresponding targets of HSHP. CHD disease targets were extracted from Genecards, OMIM, PharmGkb, TTD, and DrugBank databases. Then, the protein-protein interaction (PPI) network was constructed using the STRING web platform and Cytoscape software. GO functional and KEGG pathway enrichment analyses were carried out on the Metascape web platform. Finally, molecular docking of the active components was assessed to verify the potential targets of HSHP to treat CHD by the AutoDock Vina and PyMOL software.

**Results:**

Totally, 243 active components and 2459 corresponding targets of LDP were screened out. Eighty-five common targets of HSHP and CHD were identified. The results of the network analysis showed that resveratrol, anthranone, emodin, and ursolic acid could be defined as four therapeutic components. TNF, ESR1, NFКB1, PPARG, INS, TP53, NFКBIA, AR, PIK3R1, PIK3CA, PTGS2, and NR3C1 might be the 12 key targets. These targets were mainly involved in the regulation of biological processes, such as inflammatory responses and lipid metabolism. Enrichment analysis showed that the identified genes were mainly involved in fluid shear force, insulin resistance (IR), inflammation, and lipid metabolism pathways to contribute to CHD. This suggests that resveratrol, anthranone, emodin, and ursolic acid from HSHP can be the main therapeutic components of atherosclerosis.

**Conclusion:**

Using network pharmacology, we provide new clues on the potential mechanism of action of HSHP in the treatment of CHD, which may be closely related to the fluid shear force, lipid metabolism, and inflammatory response.

## 1. Introduction

Cardiovascular diseases, which seriously affect the quality of life of patients and bring a huge burden to the society, are the main cause of death worldwide [1–3]. Studies also showed that the incidence of cardiovascular diseases in the elderly is nearly three times higher than in other age groups [4]. Studies also showed that the incidence of cardiovascular diseases in the elderly is nearly three times higher than in other age groups [5]. The use of statins and antiplatelet agents has significantly reduced the incidence of cardiovascular events. However, the long-term risk of drug dependence and residual CHD remains an unanswered question [6]. CHD is a disease caused by multiple factors. Long-term repeated use of a certain drug may produce drug resistance. Therefore, it is very necessary to find new targets for the combined application of drugs.

The underlying mechanisms induced by the traditional Chinese medicine (TCM) are complex, and its components and targets are often multiple [7]. Clinical experience has shown that the combined application of *Polygonum cuspidatum* with detoxification effect and Hawthorn with activating blood effect can relieve the degree of angina pectoris to a certain extent. The cardiovascular activity and clinical significance of HSHP extracts have been extensively studied. *Crataegus* sp., commonly known as hawthorn, belongs to the family Rosaceae. Studies have showed that the main components of hawthorn extract, such as flavonoids, polyphenols, and oligo-procyanidins, have antiatherosclerotic effects [8]. However, its therapeutic effect on patients with CHD has not been reported. *P*. *cuspidatum* is often used in TCM to clear dampness and heat, and detoxifying, promoting blood circulation, and removing blood stasis. Moreover, *P*. *cuspidatum* is also used for its anti-inflammatory effects. In particular, resveratrol, which is main *P*. *cuspidatum* in component, was shown to play an important role in improving vasodilation and prevent thrombosis and atherosclerosis onset [9]. Our previous research confirmed that resveratrol can regulate intracellular lipid metabolism through peroxisome proliferator-activated receptors (PPARs), inhibit the formation of macrophages and foam cells, and have a certain anti-inflammatory effect in a mouse model of atherosclerosis [9]. At the same time, it has also been confirmed that flavonoids extracted from hawthorn can reduce the serum total cholesterol (TC), triglyceride (TG) and low-density lipoprotein cholesterol (LDL-C) levels and increase high-density lipoprotein cholesterol (HDL-C) levels, and at the same time, have a protective effect on aortic endothelial cells and can also reduce the size of atherosclerotic plaque [10]. However, the specific molecular mechanism triggered by the combined treatment with HSHP in CHD still needs further investigations.

In addition, due to the diversity of TCM components and the complexity of their interactions with the human body, traditional single-agent or monomer studies cannot fully explain the specific therapeutic effects of TCM [11, 12]. Therefore, we study the mechanism of HSHP in CHD by network pharmacology. Network pharmacology explains the mechanism of disease and drug action from the overall perspective of biological networks. It is an emerging discipline that is developed on the basis of system pharmacology and bioinformatics to study the interaction between drugs and disease [13]. Network pharmacology researches disease treatment targets based on the characteristics of TCM compound prescriptions and bioinformatics. By predicting the complex “drug-target-disease” relationship, it is helpful for clinical drug safety and effectiveness evaluation [14]. In addition, the multicomponent, multitarget, and regulatory network based on network pharmacology can reveal the clinically complex mechanism of action of drugs, which is particularly suitable for studying the mechanism of TCM compounds and their complex components from a “holistic perspective” [15].

**Figure 1:**
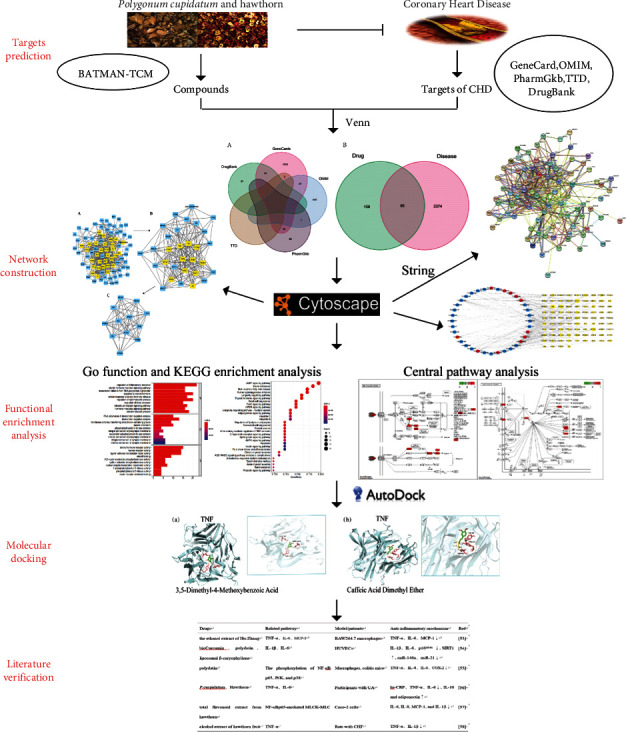
The flowchart of HSHP in treating CHD based on the network pharmacology approach.

This study uses network pharmacology and bioinformatics analysis to predict the candidate compounds and mechanisms of HSHP in the treatment of CHD. In addition, we also combined with literature analysis to clarify the relationship between the herbal active components and diseases, thereby identifying a new strategy for the prevention and treatment of CHD. The specific network pharmacology and bioinformatics approach employed in the study are shown in Figure 1.

## 2. Materials and Methods

### 2.1. Effective Chemical Composition and Targets of HSHP

BATMAN-TCM database (https://bionet.ncpsb.org/batman-tcm/) was specifically designed for the study of TCM molecular mechanisms, allowing to predict the TCM component targets and the complete component-target-pathway association network. The chemical constituents of P. *cuspate* and hawthorn were explored using the BATMAN-TCM database, as detailed in Supplementary Table S1. The Batman-TCM database, with a score cut-off of 20 and an adjusted *P*-value of 0.05 (as shown in Supplementary Table S2), was used to search for active components of HSHP, and their corresponding disease targets.

### 2.2. Potential Disease Target Genes

UniProt identifiers of HSHP were obtained from the UniProt database (https://www.un.org). GeneCard (https://www.genecards.org/), OMIM (https://www.omim.org/), PharmGkb (https://www.pharmgkb.org/), Therapeutic Targets Database (https://db.idrblab.net/ttd/), and DrugBank (https://www.drugbank.ca) were used to search CHD-related genes. Venny v2.1 (https://bioinfogp.cnb.csic.es/tools/venny/index.html) online tool was used to map the drug-diseases overlap genes. The common targets of HSHP and CHD could represent the potential therapeutic targets. Finally, Cytoscape software (Version 3.7.1) was used to map the disease network of drug components.

### 2.3. “Herbal-Compound-Target” Network Construction

The “Herbal-compound-target” network was constructed using the network visualization software Cytoscape (Version 3.7.1). The network diagram of proteins and their interactome was also constructed. Topological parameters were used to screen the key active compounds and CHD-related targets of HSHP. The String database (https://string-db.org/cgi/input.pl) was used to explore the PPI. The “organism” option was set to “*Homo sapiens,*” and PPI with a composite score greater than 0.5 was selected as the core gene.

### 2.4. Protein-Protein Interaction (PPI) Network and Key Gene Screening

The CHD targets related to HSHP obtained from the String database were mapped using Cytoscape software (Version 3.7.1). The species parameter was set as “*Homo sapiens*,” and the confidence score was limited to “>0.7,” hide discrete targets, the PPI network was built, and the date of the network was exported. We then used the Cytoscape plugin CytoNCA to screen critical targets in the PPI network. Betweenness centrality, closeness centrality, and degree centrality were chosen as the parameters to calculate topological features of the PPI network. Betweenness centrality was used to assess how much the shortest paths must pass through a given node.

### 2.5. Enrichment Analysis

In order to further understand the underlying mechanisms of HSHP for the treatment of CHD, gene ontology (GO, https://geneontology.org/), function annotation, and the Kyoto Encyclopedia of Genes and Genomes (KEGG, https://www.genome.jp/kegg/) pathway enrichment analysis were performed on the 85 common target genes of the identified drug-disease-target network using the R 3.6.1 software with the Bioconductor Package. The obtained target genes molecular function (MF), biological process (BP), and cellular component (CC) were analysed. *P*-value <0.05 was considered statistically significant. The top 30 items with the highest enrichment were selected for analysis. Lastly, the RGUI and pathview tools in the KEGG database were used to refine target list and identify the top 20 genes. The target pathway network was established through the connection between the target and KEGG pathway analysis data, and the pathway with a degree greater than or equal to the median, and related to CHD, was selected as the main pathway for further research.

### 2.6. Acquisition of Drug-Like Components and CHD Target Crystals and Molecular Docking Verification

The drug components of HSHP, and the targets of CHD, were obtained by analysing the drug-disease-target network. The chemical library database (https://www.chemicalbook.com/) was used to find and download the drug molecular structure. The protein crystal structure of the CHD-related target was obtained from the RCSB Protein Data Bank database (https://www.rcsb.org/). The structure and chemical formula of the target and drug, respectively, were obtained from the RCSB Protein Data Bank structured and PubChem (https://pubchem.ncbi.nlm.nih.gov/). The AutoDock Tools 1.5.6 (https://autodock.scripps.edu) was used to perform hydrogenation and remove water molecules for protein processing and molecular docking and calculate the docking binding energy of each complex to evaluate the binding effect. PyMol 2.3.2 software (https://pymol.org/2/) was used to predict the protein molecular docking mechanism.

## 3. Results

### 3.1. Active Components and Disease Targets of HSHP

Active components of HSHP, and their corresponding disease targets, were acquired from the Batman-TCM database (as shown in Supplementary Table S2). A total of 85 active components were selected (the results are shown in Supplementary Table S3). Resveratrol, a polyphenolic compound extracted from the root of P. *cuspidatum*, has been proved to have antiatherosclerosis, immune regulatory [16], antibacterial [17], and anti-inflammatory pharmacological effects [18]. Emodin was extracted from the dried rhizome and root of P. *cuspidatum* and was shown to inhibit the levels of inflammatory factors and reactive oxygen species (ROS) by inhibiting the TLR4/NF-*κ*B signalling pathway, thereby acting as an anti-inflammatory and antioxidant agent, protecting the vascular endothelium and preventing atherosclerosis [19]. Ursolic acid is a pentacyclic triterpene and was demonstrated to have anti-inflammatory, antitumour, lipid-lowering, and other pharmacological activities [20].

### 3.2. “Herbal-Compound-Target” Network Analysis

**Figure 2:**
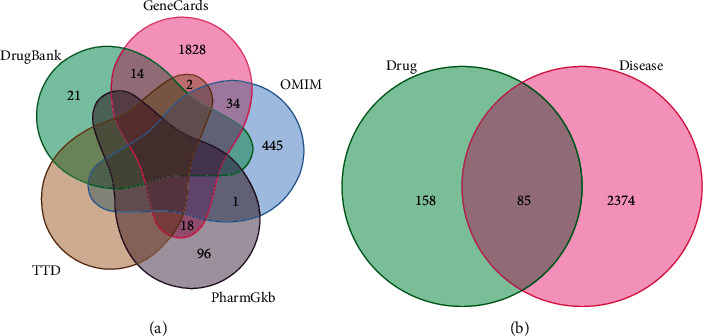
Venn diagram of the HSHP targets. (a) CHD disease targets. (b) The intersection of HSHP and CHD disease targets.

**Figure 3:**
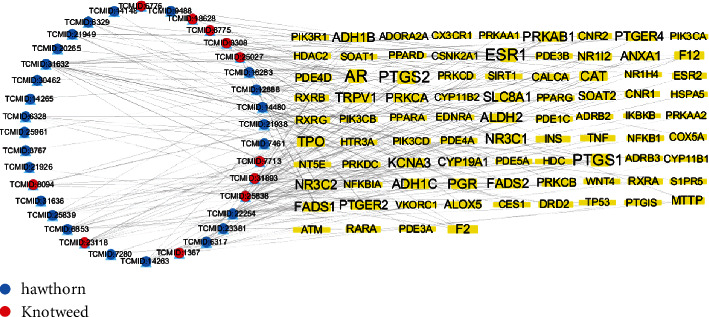
Network diagram of interaction between *Polygonum cuspidatum* and hawthorn and the target disease (red and blue knots represent the main active components of HSHP, respectively, and the yellow knots represent their potential targets in the treatment of CHD).

The effective active component targets collected from UniProt database were merged with the disease genes collected from Genecards (1,896 compounds), OMIM (480 compounds), PharmGkb (115 compounds), TTD (2 compounds), and DrugBank (35 compounds) (Figure 2(a)). Among them, there were 243 active components corresponding targets, 2,459 disease targets, and 85 intersections genes (Figure 2(b)). Active components and target genes were entered into the Cytoscape software, then the isolated components were deleted because of the lack of intersection with the targets. And we acquire a network diagram of the interaction between the drug component and target disease (Figure 3). The degree value represented the number of associations between the component and the target. The greater the degree value of the target point, the more important is its component.

### 3.3. PPI Network and Key Targets Analysis

**Figure 4:**
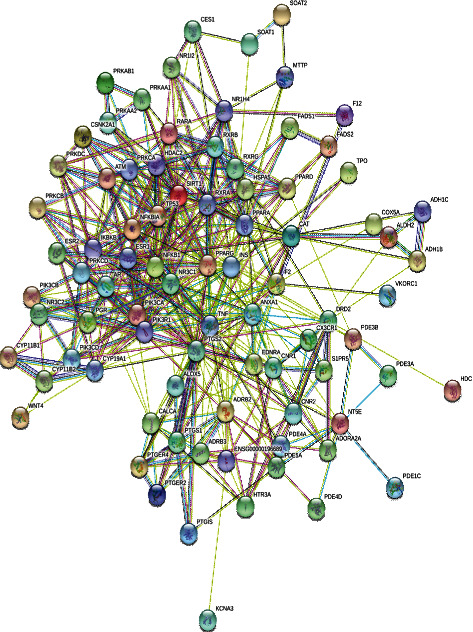
PPI network of HSHP and CHD common targets.

**Figure 5:**
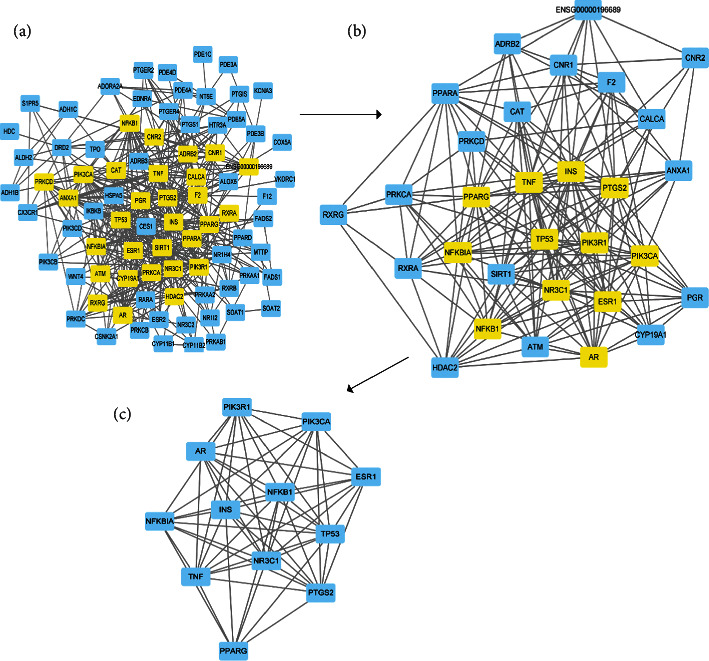
Screening of the key targets in the PPI network.

We introduced 85 common targets into the STRING online service platform, and the date and figure of a PPI network were acquired (Figure 4). Cytoscape software (Version 3.7.1) was used to construct the component-target network, and CytoNCA was used to obtain the PPI network after two screenings. In total, 30 nodes were obtained in the first screening, and 12 targets (INS, PPARG, TNF, NR3C1, TP53, PIK3CA, PIK3R1, NFKB1, NFKBIA, PTGS2, ESR1, and AR) were finally obtained after a second screening (Figure 5). The PPI network nodes represent the proteins, and the edges represent the interactions between the proteins.

### 3.4. GO and KEGG Pathways Enrichment and Target Path Analyses

**Figure 6:**
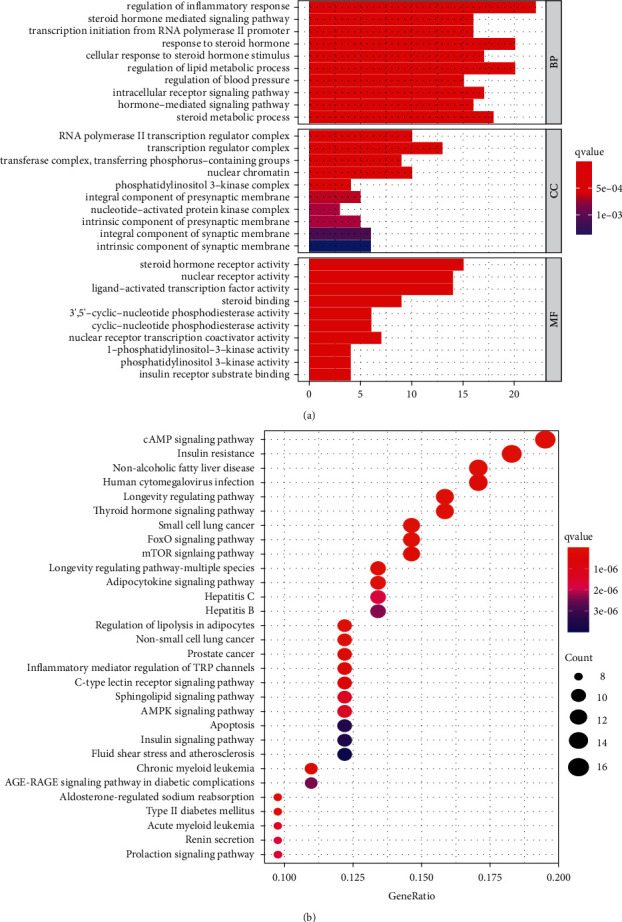
GO functional and KEGG pathway enrichment analyses of HSHP potential therapeutic targets for CHD. The top 30 terms of BP, CC, MF in GO functional and KEGG terms were identified based on the main active ingredients of HSHP (BP: biological processes; CC: cellular component; MF: molecular function).

**Figure 7:**
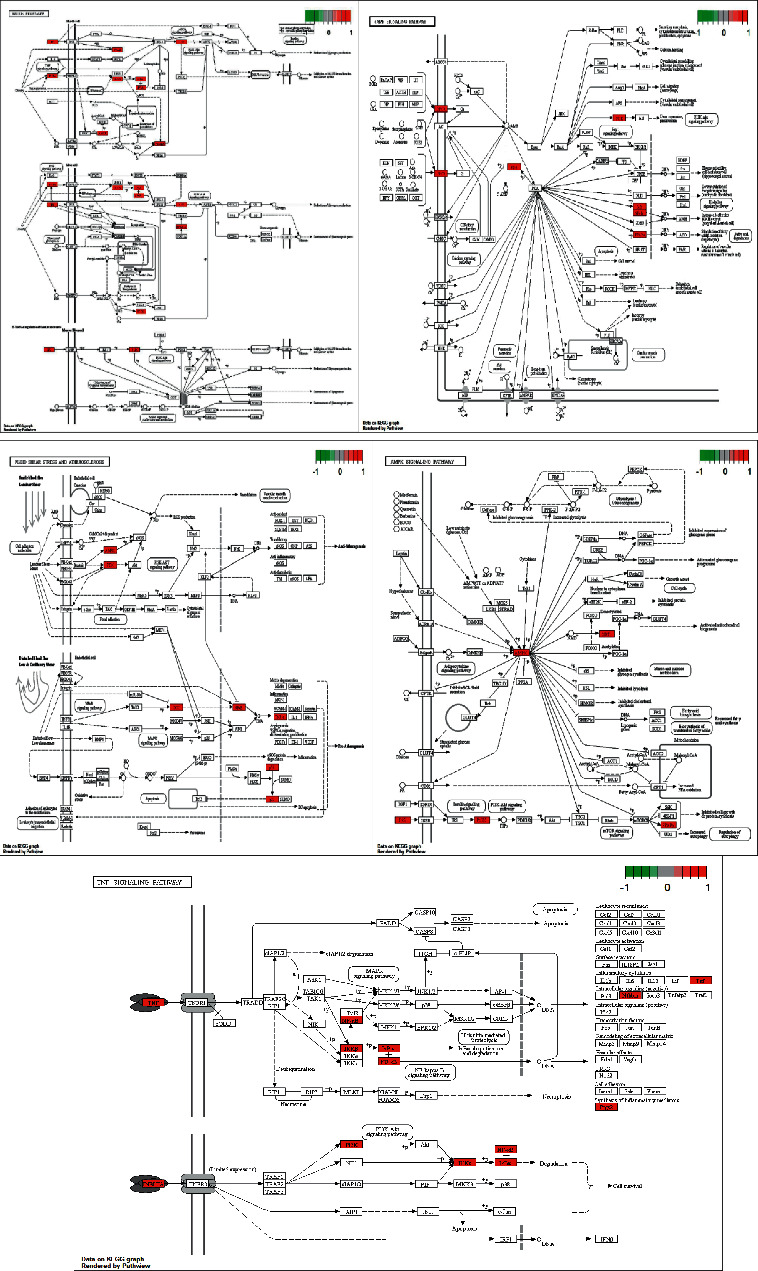
Signalling pathway in CHD.

Using the DAVID database, the MF, BP, and CC of the 58 core targets in the treatment of CHD were analysed, and 81 molecular functions were obtained. With *P*-value <0.05 as the threshold, the top 30 GO biological function analysis results were screened out (Figure 6(a)). Among them, the most relevant BPs were the regulation of inflammatory response and blood pressure, regulation of lipid and steroid metabolism, intracellular and hormone-mediated signalling pathways, and other metabolic aspects. The same method was used for the KEGG enrichment analysis, and the relevant signalling pathways were obtained, using *P*-value <0.05 as the threshold. The top 30 KEGG results were mainly composed of insulin, longevity regulation, cAMP, mTOR, and AMPK signalling pathways. Various types of apoptosis and fluid shear stress elements were also identified (Figure 6(b)). The signalling pathway maps of HSHP for the treatment of CHD were obtained by the KEGG Mapper tool (Figure 7; Supplementary Table S6).

### 3.5. Molecular Docking Analysis

**Figure 8:**
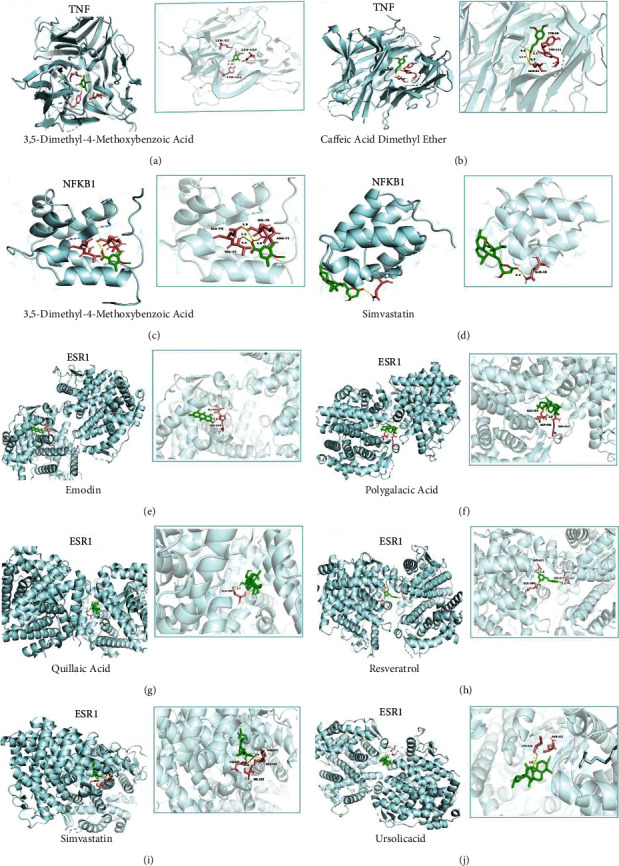
Detailed target compound interactions in the docking simulation. (a) TNF protein ginsenoside 3,5-dimethyl-4-methoxybenzoic acid. (b) TNF protein ginsenoside caffeic acid dimethyl ether. (c) NF-*κ*B1 protein ginsenoside 3,5-dimethyl-4-methoxybenzoic acid. (d) NF-*κ*B1protein ginsenoside simvastatin. (e) ESR1 protein ginsenoside emodin. (f) ESR1 protein ginsenoside polygalacic acid. (g) ESR1 protein ginsenoside quillaic acid. (h) ESR1 protein ginsenoside resveratrol. (i) ESR1 protein ginsenoside simvastatin. (j) ESR1 protein ginsenoside ursolicacid.

For the purpose of validating the study results of the network analysis, the molecular docking between the key targets (TNF, NF-*κ*B, and ESR1) and their corresponding active compounds was performed. Discovery Studio software was used to observe the compounds entering the protein active pocket, and their affinity (Kcal/mol) was used to identify the degree of ligand binding to the receptor protein and verify the therapeutic mechanism of hawthorn in the treatment of CHD (the results are shown in Supplementary Table S5). The drug simvastatin was used as control in the analysis. When the binding energy is <0 kJmol, the small molecule ligand can spontaneously bind to the protein receptor. If the binding energy is <−5.0 kJmol or lower, it indicates that the two have the better binding ability. No significant difference in the molecular docking matching between HSHP was observed. The results showed that there was no significant difference between the molecular docking of HSHP and simvastatin, which validated the results of the network pharmacology analysis. These findings also verified the regulatory effects of HSHP on the targets NF-*κ*B and ESR1 of CHD. The docking results of HSHP to the CHD protein receptor were shown in Supplementary Table S4, and the partial best molecular docking target processes and the bnding free energy are shown in Figure 8 and Table 1.

## 4. Discussion

Atherosclerosis is one of the major causes of CHD worldwide [27]. Currently, the toxic and side effects of commonly used synthetic drugs, such as statins, nicotine, angiotensin receptor blockers, antioxidants, anti-platelets, and anticoagulants, limit their use in the treatment of CHD [28]. Chinese herbal medicine is often a multicomponent, multitarget, and low-cost therapeutic option that does not have obvious toxic and side effects. According to previous investigations, there are relevant evidences that the compatibility of hawthorn and *P. cuspidatum* may hold potential for the treatment of atherosclerosis [9, 10], and possibly CHD. Therefore, this study adopted the network pharmacology analysis approach to comprehensively assess the underling mechanism of action of HSHP in the treatment of CHD.

Herein, NF-*κ*B, TNF, and ESR1 were found to be hub targets based on the results of the network analysis. NF-*κ*B is a protein complex found in various animal cells and is involved in the cellular response to stimuli. The main function of NF-*κ*B is to initiate gene transcription. Inflammatory mediators and cytokines are one of the transcription products regulated by NF-*κ*B. NF-*κ*B plays an important role in the occurrence and development of CHD by regulating the transcription of downstream inflammatory mediators and cytokines [29]. Jin et al. [30] showed that NF-*κ*B1 plays an important role in the regulation of inflammation, and mutation of its coding gene is related to the risk of acute coronary syndrome among han people in Xinjiang, China [31]. In addition, omega 3 fatty acids reduce NF-*κ*B1, thereby altering the endothelial cell function, reducing inflammation, and slowing the development of atherosclerosis. Studies have shown that resveratrol can protect the cardiovascular tissues of rats with CHD and diabetes by downregulating the TLR4/MyD88/NF-*κ*B signalling pathway [32]. TNF-*α* is produced by monocytes and macrophages, exists in atherosclerotic plaques, and participates in the formation of atherosclerosis. TNF-*α* levels are elevated in patients with CHD, which can cause myocardial remodeling and aggravate heart function. Inflammatory factors IL-1 and TNF-*α* are regulated by NF-*κ*B. Inflammatory factors IL-1 and TNF-*α* are regulated by NF-*κ*B. At the same time, IL-1, TNF-*α* and related cytokines released form a positive and negative feedback loop that can activate NF-*κ*B and cause cytokines to continuously increase. Participate in the occurrence and development of atherosclerosis. Clinical studies have shown that the interaction between TNF-*α* and oxidative stress is related to the severity of coronary atherosclerosis and can be used as a potential noninvasive diagnostic organism for coronary chronic total occlusions (CCTO) in elderly patients with CHD landmark [33]. Studies have shown that oestrogen can inhibit the development of atherosclerosis and has a direct antiatherosclerosis effect [34, 35]. Moreover, when atherosclerosis occurs, the early use of oestrogen therapy can allow oestrogen to fully bind to its receptor, thus protecting the blood vessels [36, 37]. Therefore, inflammation and oestrogen receptor are likely to be important target pathways for HSHP to treat atherosclerosis.

In order to further understand the mechanism of HSHP against atherosclerosis, the GO biological function analysis and the KEGG functional enrichment analysis was performed. The GO biological function analysis revealed that the BP mainly involved were the regulation of inflammatory response, fat and steroid metabolic processes, blood pressure, as well as hormone-mediated and intracellular receptor signalling pathways. MF mainly focused on lipid- and inflammation-related aspects, such as steroid receptor activity, steroid binding, and nuclear receptor activity. CC was mainly concentrated in the transcription regulator complex, RNA polymerase II transcription regulator complex, nuclear chromatin, and other aspects. The KEGG analysis results showed that the therapeutic approaches of HSHP against atherosclerosis were mainly related to hemodynamic, metabolic disorders, and inflammatory signalling pathways, including fluid shear force and IR, as well as AMPK, cAMP, and mTOR signalling pathways. These findings are in agreement with our previous studies, which demonstrated that cAMP, mTOR, and AMPK pathways play key roles in the therapeutic mechanism of atherosclerosis by regulating inflammation, lipid metabolism, cell proliferation, and apoptosis.

The results showed that resveratrol, anthranone, emodin, and ursolic acid were the most probable therapeutic components identified by the composition-to-target network and subsequent molecular docking affinity analysis (Table 1). Modern pharmacology has showed that resveratrol has anti-inflammatory, anti-tumour, and other biological effects [38]. Xiong et al. [39] found that by inhibiting the activation of the PI3K/Akt/mTOR pathway in ApoE^−/−^ mice, resveratrol could play an antiatherosclerosis role. Emodin is an anthraquinone compound, which have been shown to have anti-inflammatory and cardiovascular protective effects [40–42]. Nemmar et al. [43] found that emodin can prevent the release of TNF induced by diesel exhaust particles, significantly alleviating the changes in the activity of the antioxidant enzymes superoxide dismutase and glutathione reductase E, reducing the risk of thrombus formation in human arterioles and venules, thereby protecting the cardiovascular system. Moreover, Seo et al. [44] found that emodin-8-o-glucoside inhibits platelet aggregation induced by collagen and thrombin, significantly prolonging bleeding time *in vivo*. Other studies have shown that aloe emodin can promote retinal neovascularization by inhibiting the HIF-1/VEGF signalling pathway, possibly playing a role in the treatment of diabetic retinopathy [45]. Ursolic acid and its isomer oleanolic acid are pentacyclic triterpenes derived from plants, which have antitumour, antiliver fibrosis, antiatherosclerosis, and other effects [46]. Studies showed that ursolic acid, oleanolic acid, and their derivatives can inhibit the activation of proinflammatory pathways and promote the transcription of antioxidants by activating Nrf2, thus exerting anti-inflammatory and antioxidant effects [47]. Therefore, resveratrol, anthranone, emodin, and ursolic acid may be important components in the treatment of CHD.

Based on the previous related research of our team and other literature, we further studied the prediction results of network pharmacology. Our previous research results showed that HSHP may inhibit the activation of NF-*κ*B, ESR1, and TNF signalling pathways and downregulate the expression of inflammatory factors IL-6, mainly by reducing serum lipid levels such as TG, TC, ApoB100, and Lp(a) [10]. These results support the network pharmacology data and prove that HSHP affects the expression of core genes and changes the signalling pathways such as NF-*κ*B, ESR1, and TNF. TNF is an important proinflammatory factor that mediates inflammatory and immune responses. It can regulate lipid metabolism and stimulate the expression of adhesion molecules in itself, and IL-1 and IL-6 cells play a pro-inflammatory effect and accelerate the formation of atherosclerosis [48]. Some studies have reported that resveratrol and its analogs may inhibit the early events of atherosclerosis by regulating the adhesion and transport of monocytes [49]. Resveratrol can significantly reduce the plasma and liver TG, TC, and free fatty acid concentrations in high fat diet rats, and the mechanism of its action may be related to the reduction of liver TNF-*α* expression and lipid peroxidation levels [50]. In a mice model of ulcerative colitis, polydatin targets the NF-*κ*B-p65 pathway and exerts an anti-inflammatory effect by blocking the expression of the main inflammatory cytokines TNF-*α*, IL-6, and IL-1*β* [51]. In addition, studies have observed that the beneficial effect of polydatin on HFD-induced obese mice is attributed to the regulation of the expression of TNF-*α*, MCP-1, IL-6, S100A8, and S100A9 triggered inflammation [52]. In this study, in addition to verifying the TNF signalling pathway predicted by the PPI target gene, our previous research also found that the downstream IL-6 inflammatory factor expression changes. The related literature was shown in Table 2.

However, contrasting with previously reported data, these results also suggested that shear force and IR could be involved in the antiatherosclerotic mechanisms induced by HSHP. The turbulence and low shear stress generated at the bifurcation, branching exit, and bending of blood vessels may promote cell proliferation and apoptosis, promoting the inflammatory response, lipid uptake, and synthesis by the endothelium, as well as the subcutaneous aggregation of monocytes and lipids, thereby increasing the risk of plaque and thrombus formation [53]. In addition, temporary activation of NF-*κ*B in straight arteries under normal endothelial shear stress would play an important biological role in improving eNOS expression, as well as the expression of its target genes, such as MCP-1, ICAM-1, and VCAM-1, which will in turn promote white blood cell aggregation and the inflammatory response.

IR is the main line and central link of various metabolic abnormalities and CHD [54] and is closely related to the occurrence and development of CHD. For example, studies have shown the presence of IR in patients with CHD [55]. Moreover, IR can cause and accelerate atherosclerosis through inflammation, oxidative stress reaction, lipid metabolism disorder, endothelial injury, and other factors. IR can activate JNK and IKK by increasing the levels of ROS to initiate and amplify the inflammatory response and promote the occurrence of endothelial disorders [56]. For example, JNK promotes the secretion of inflammatory molecules such as matrix metals proteins, interleukin-2 (IL-2), and TNF-*ɑ* [57]. In turn, TNF-*ɑ* and IL-6 can affect the insulin signal transduction and increase the release of free fatty acids (FFA), activates the MAPK pathway, endothelial DAG and PKC signal pathways, and intimal smooth muscle cells from middle to vascular endothelial migration and activation, as well as the synthesis and secretion of extracellular matrix proteins and fibrinolytic enzyme activator inhibitor 1 that will promote thrombosis [58].

## 5. Conclusion

This study predicted the targets, mechanism, and related signal pathways of HSHP in the treatment of CHD through network pharmacology, verified the binding abilities of herb active ingredients and their targets. Through molecular docking, this study found that the docking effect of the common compounds of HSHP, including resveratrol, emodin, and ursolic acid, was similar to that of simvastatin. And these active components bind well with the key targets NF-*κ*B, TNF, and ESR1. Therefore, it is possible that HSHP plays an important role in the treatment of atherosclerosis, and their therapeutic mechanism is mainly mediated by the modulation of hub targets involved in blood flow shear force, IR, inflammation, and metabolism-related signalling pathways. However, the study also has some limitations, including the investigation of biologically active ingredients and further experimental verification to verify the impact of HSHP on CHD. Therefore, further research is needed to demonstrate HSHP potential therapeutic mechanisms.

## Figures and Tables

**Table 1:** Key components and target molecular docking information of HSHP.

Targets	Protein	Ingredient	Id	Binding free energy (kcal/mol)
TNF	TNF	3,5-Dimethyl-4-methoxybenzoic acid	TCMID:25838	−7.8
TNF	TNF	Caffeic acid dimethyl ether	TCMID:23381	−7.6
NF-*κ*B	NF-*κ*B	3,5-Dimethyl-4-methoxybenzoic acid	TCMID:25838	−4.5
NF-*κ*B	NF-*κ*B	Simvastatin	CID:54454	−6.4
ESR1	ESR1	Emodin	TCMID:6775	−8.2
ESR1	ESR1	Polygalacic acid	TCMID:25027	−7.4
ESR1	ESR1	Quillaic acid	TCMID:31893	−7.9
ESR1	ESR1	Resveratrol	TCMID:18628	−7.2
ESR1	ESR1	Simvastatin	CID:54454	−9.3
ESR1	ESR1	Ursolic acid	TCMID:22254	−7.9

**Table 2:** HSHP exerts anti-inflammatory effects on atherosclerosis via inhibiting NF-*κ*B, TNF, and IL-6 signalling pathways.

Drugs	Related pathway	Model/patients	Anti-inflammatory mechanism	Ref
The ethanol extract of hu-zhang	TNF-*α*, IL-6, MCP-1	RAW264.7 macrophages	TNF-*α*, IL-6, MCP-1↓	[21]
bioCurcumin, polydatin, liposomal *β*-caryophyllene	IL-1*β*, IL-6	HUVECs	IL-1*β*, IL-6, p16^ink4a^ ↓, SIRT1↑, miR-146a, miR-21↓	[22]
Polydatin	The phosphorylation of NF-*κ*B p65, JNK, and p38	Macrophages, colitis mice	TNF-*α*, IL-4, IL-6, COX-2↓	[23]
*P*. *cuspidatum*, hawthorn	TNF-*α*, IL-6	Participants with UA	hs-CRP, TNF-*α*, IL-6↓, IL-10, and adiponectin↑	[24]
Total flavonoid extract from hawthorn	NF-*κ*Bp65-mediated MLCK-MLC	Caco-2 cells	IL-6, IL-8, MCP-1, and IL-1*β*↓	[25]
Alcohol extract of hawthorn fruit	TNF-*α*	Rats with CHF	TNF-*α*, IL-1*β*↓	[26]

HUVECs: human umbilical vein endothelial cells; JNK: c-Jun N-terminal kinase; COX-2: cyclooxygenase-2; CHF: chronic heart failure; UA: unstable angina.

## Data Availability

The data used to support the findings of this study are included within the article and the supplementary materials.

## List of Abbreviations

BP: Biological process
*Crataegus sp.*: Hawthorn (Shanzha)
CHD: Coronary heart disease
CHF: Chronic heart failure
CCTO: Coronary chronic total occlusions
COX-2: Cyclooxygenase-2
CC: Cellular component
FFA: Free fatty acids
HSHP: Huzhang–Shanzha herb pair
HDL-C: High-density lipoprotein cholesterol
HUVECs: Human umbilical vein endothelial cells
IR: Insulin resistance
IL-2: Interleukin-2
JNK: c-Jun N-terminal kinase
KEGG: Kyoto encyclopedia of genes and gnomes
LDL-C: Low-density lipoprotein cholesterol
MF: Molecular function
PPARs: Peroxisome proliferator-activated receptors
PPI network: Protein-protein interaction network
P. *cuspidatum*: *Polygonum cuspidatum* (huzhang)
ROS: Reactive oxygen species
TCM: Traditional Chinese medicine
TC: Total cholesterol
TG: Triglyceride
TTD: Therapeutic target database
UA: Unstable angina

## Supplementary Materials

Supplementary Materials Supplementary Table S1: The information of all ingredients of HSHP for coronary heart disease. Supplementary Table S2: Active ingredient parameters of HSHP. Supplementary Table S3: All genes of HSHP in the treatment of CHD. Supplementary Table S4: Compound-target pair information. Supplementary Table S5: Results of ligand-receptor protein molecular docking. Supplementary Table S6: signalling pathway in CHD.

## References

[1] Virani S. S., Alonso A., Benjamin E. J. (2020). Heart disease and stroke statistics-2020 update: a report from the american heart association. *Circulation*.

[2] Benjamin E. J., Muntner P., Alonso A. (2019). Heart disease and stroke statistics-2019 update: a report from the american heart association. *Circulation*.

[3] Xu S., Xu Y., Liu P. (2019). The novel coronary artery disease risk gene JCAD/KIAA1462 promotes endothelial dysfunction and atherosclerosis. *European Heart Journal*.

[4] Finegold J. A., Asaria P., Francis D. P. (2013). Mortality from ischaemic heart disease by country, region, and age: statistics from world health organisation and United Nations. *International Journal of Cardiology*.

[5] Tabas I., García-Cardeña G., Owens G. K. (2015). Recent insights into the cellular biology of atherosclerosis. *Journal of Cell Biology*.

[6] HPS2-THRIVE Collaborative Group, Haynes R., Jiang L. (2013). HPS2-THRIVE randomized placebo-controlled trial in 25 673 high-risk patients of ER niacin/laropiprant: trial design, pre-specified muscle and liver outcomes, and reasons for stopping study treatment. *European Heart Journal*.

[7] Zhang Q., Fu X., Wang J., Yang M., Kong L. (2017). Treatment effects of ischemic stroke by berberine, baicalin, and jasminoidin from huang-lian-jie-du-decoction (HLJDD) explored by an integrated metabolomics approach. *Oxidative Medicine and Cellular Longevity*.

[8] Wu M., Liu L., Xing Y., Yang S., Li H., Cao Y. (2020). Roles and mechanisms of hawthorn and its extracts on atherosclerosis: a review. *Frontiers in Pharmacology*.

[9] Liu L. T., Guo G., Wu M., Zhang W. G. (2012). The progress of the research on cardio-vascular effects and acting mechanism of polydatin. *Chinese Journal of Integrative Medicine*.

[10] Wu M., Liu M., Guo G., Zhang W., Liu L. (2015). Polydatin inhibits formation of macrophage-derived foam cells. *Evidence Based Complementary Alternative Medicine*.

[11] Hung Y. C., Wang P. W., Pan T. L. (2010). Functional proteomics reveal the effect of *Salvia miltiorrhiza* aqueous extract against vascular atherosclerotic lesions. *Biochimics et Biophysica Acta*.

[12] Novakovic A., Marinko M., Jankovic G. (2017). Endothelium-dependent vasorelaxant effect of procyanidin B2 on human internal mammary artery. *European Journal of Pharmacology*.

[13] Escala-Garcia M., Abraham J., Andrulis I. L. (2020). A network analysis to identify mediators of germline-driven differences in breast cancer prognosis. *Nature Communications*.

[14] Lee W. Y., Lee C. Y., Kim Y. S., Kim C. E. (2019). The methodological trends of traditional herbal medicine employing network pharmacology. *Biomolecules*.

[15] Moodley D., Yoshida H., Mostafavi S. (2016). Network pharmacology of JAK inhibitors. *Proceedings of the National Academy of Sciences*.

[16] Zhou L., Long J., Sun Y., Chen W., Qiu R., Yuan D. (2020). Resveratrol ameliorates atherosclerosis induced by high-fat diet and LPS in ApoE−/− mice and inhibits the activation of CD4+ T cells. *Nutrition & Metabolism*.

[17] Kumar S., Chang Y. C., Lai K. H., Hwang T. L. (2020). Resveratrol, a molecule with anti-inflammatory and anti-cancer activities: natural product to chemical synthesis. *Current Medicinal Chemistry*.

[18] Mota M., Porrini V., Parrella E. (2020). Neuroprotective epi-drugs quench the inflammatory response and microglial/macrophage activation in a mouse model of permanent brain ischemia. *Journal of Neuroinflammation*.

[19] Luo S., Deng X., Liu Q. (2018). Emodin ameliorates ulcerative colitis by the flagellin-TLR5 dependent pathway in mice. *International Immunopharmacology*.

[20] You H. J., Choi C. Y., Kim J. Y., Park S. J., Hahm K.-S., Jeong H. G. (2001). Ursolic acid enhances nitric oxide and tumor necrosis factor-*α* production via nuclear factor-*κ*B activation in the resting macrophages. *FEBS Letters*.

[21] Yu M., Chen T. T., Zhang T. (2021). Anti-inflammatory constituents in the root and rhizome of *Polygonum cuspidatum* by UPLC-PDA-QTOF/MS and lipopolysaccharide-activated RAW264.7 macrophages. *Journal of Pharmaceutical and Biomedical Analysis*.

[22] Matacchione G., Gurău F., Silvestrini A. (2021). Anti-SASP and anti-inflammatory activity of resveratrol, curcumin and *β*-caryophyllene association on human endothelial and monocytic cells. *Biogerontology*.

[23] Chen G., Yang Z., Wen D. (2021). Polydatin has anti‐inflammatory and antioxidant effects in LPS‐induced macrophages and improves DSS‐induced mice colitis. *Immunity, Inflammation and Disease*.

[24] Wu M., Yang S., Liu G. (2021). Treating unstable angina with detoxifying and blood-activating formulae: a randomized controlled trial. *Journal of Ethnopharmacology*.

[25] Liu F., Zhang X., Ji Y. (2020). Total flavonoid extract from hawthorn (Crataegus pinnatifida) improves inflammatory cytokines-evoked epithelial barrier deficit. *Medical Science Monitor: International Medical Journal of Experimental and Clinical Research*.

[26] Cheng F., Jiang W., Xiong X., Chen J., Xiong Y., Li Y. (2020). Ethanol extract of Chinese hawthorn (Crataegus pinnatifida) fruit reduces inflammation and oxidative stress in rats with doxorubicin-induced chronic heart failure. *Medical Science Monitor: International Medical Journal of Experimental and Clinical Research*.

[27] Nettersheim F. S., de Vore L., Winkels H. (2020). Vaccination in atherosclerosis. *Cells*.

[28] Siasos G., Tsigkou V., Kosmopoulos M. (2018). Mitochondria and cardiovascular diseases-from pathophysiology to treatment. *Annals of Translational Medicine*.

[29] Guo F., Tang C., Li Y. (2018). The interplay of Lnc RNA ANRIL and miR‐181b on the inflammation‐relevant coronary artery disease through mediating NF ‐*κ*B signalling pathway. *Journal of Cellular and Molecular Medicine*.

[30] Jin S. Y., Luo J. Y., Li X. M. (2019). NFKB1 gene rs28362491 polymorphism is associated with the susceptibility of acute coronary syndrome. *Bioscience Reports*.

[31] Luo J. Y., Li X. M., Zhou Y. (2017). Mutant DD genotype of NFKB1 gene is associated with the susceptibility and severity of coronary artery disease. *Journal of Molecular and Cellular Cardiology*.

[32] Huo X., Zhang T., Meng Q., Li C., You B. (2019). Resveratrol effects on a diabetic rat model with coronary heart disease. *Medical Science Monitor*.

[33] Li X., Zhang F., Zhou H. (2020). Interplay of TNF-*α*, soluble TNF receptors and oxidative stress in coronary chronic total occlusion of the oldest patients with coronary heart disease. *Cytokine*.

[34] Nakamura Y., Suzuki T., Miki Y. (2004). Estrogen receptors in atherosclerotic human aorta: inhibition of human vascular smooth muscle cell proliferation by estrogens. *Molecular and Cellular Endocrinology*.

[35] Hodgin J. B., Krege J. H., Reddick R. L., Korach K. S., Smithies O., Maeda N. (2001). Estrogen receptor *α* is a major mediator of 17*β*-estradiol’s atheroprotective effects on lesion size in Apoe-/- mice. *Journal of Clinical Investigation*.

[36] Clarkson T. B., Appt S. E. (2005). Controversies about HRT-lessons from monkey models. *Maturitas*.

[37] Kim G. H., Ryan J. J., Archer S. L. (2013). The role of redox signaling in epigenetics and cardiovascular disease. *Antioxidants & Redox Signaling*.

[38] Das S., Das D. K. (2007). Resveratrol: a therapeutic promise for cardiovascular diseases. *Recent Patents on Cardiovascular Drug Discovery*.

[39] Xiong Q., Yan Z., Liang J. (2020). Polydatin alleviates high-fat diet induced atherosclerosis in apolipoprotein E-deficient mice by autophagic restoration. *Phytomedicine*.

[40] Ye M., Han J., Chen H., Zheng J., Guo D. (2007). Analysis of phenolic compounds in rhubarbs using liquid chromatography coupled with electrospray ionization mass spectrometry. *Journal of the American Society for Mass Spectrometry*.

[41] Ren G., Li L., Hu H., Li Y., Liu C., Wei S. (2016). Influence of the environmental factors on the accumulation of the bioactive ingredients in Chinese rhubarb products. *PLoS One*.

[42] Sun H., Luo G., Chen D., Xiang Z. (2016). A comprehensive and system review for the pharmacological mechanism of action of rhein, an active anthraquinone ingredient. *Frontiers in Pharmacology*.

[43] Nemmar A., Al Dhaheri R., Alamiri J. (2015). Diesel Exhaust particles induce impairment of vascular and cardiac homeostasis in mice: ameliorative effect of emodin. *Cellular Physiology and Biochemistry*.

[44] Seo E. J., Ngoc T. M., Lee S. M., Kim Y. S., Jung Y. S. (2012). Chrysophanol-8-O-glucoside, an anthraquinone derivative in rhubarb, has antiplatelet and anticoagulant activities. *Journal of Pharmacological Sciences*.

[45] Wu J., Ke X., Wang W. (2016). Aloe-emodin suppresses hypoxia-induced retinal angiogenesis via inhibition of HIF-1*α*/VEGF pathway. *International Journal of Biological Sciences*.

[46] Rao A. R., Veeresham C., Asres K. (2013). In vitro and in vivo inhibitory activities of four Indian medicinal plant extracts and their major components on rat aldose reductase and generation of advanced glycation endproducts. *Phytotherapy Research*.

[47] Pitha-Rowe I., Liby K., Royce D., Sporn M. (2009). Synthetic triterpenoids attenuate cytotoxic retinal injury: cross-talk between Nrf2 and PI3K/AKT signaling through inhibition of the lipid phosphatase PTEN. *Investigative Opthalmology & Visual Science*.

[48] Fadaei R., Moradi N., Kazemi T. (2019). Decreased serum levels of CTRP12/adipolin in patients with coronary artery disease in relation to inflammatory cytokines and insulin resistance. *Cytokine*.

[49] Deng Y. H., Alex D., Huang H. Q. (2011). Inhibition of TNF-*α*-mediated endothelial cell-monocyte cell adhesion and adhesion molecules expression by the resveratrol derivative, trans-3,5,4′-trimethoxystilbene. *Phytotherapy Research: PTR*.

[50] Zhang J., Tan Y., Yao F., Zhang Q. (2012). Polydatin alleviates non-alcoholic fatty liver disease in rats by inhibiting the expression of TNF-*α* and SREBP-1c. *Molecular Medicine Reports*.

[51] Yao J., Wang J. Y., Liu L. (2011). Polydatin ameliorates DSS-induced colitis in mice through inhibition of nuclear factor-kappaB activation. *Planta Medica*.

[52] Zhao X. J., Yu H. W., Yang Y. Z. (2018). Polydatin prevents fructose-induced liver inflammation and lipid deposition through increasing miR-200a to regulate Keap1/Nrf2 pathway. *Redox Biology*.

[53] Chatzizisis Y. S., Coskun A. U., Jonas M., Edelman E. R., Feldman C. L., Stone P. H. (2007). Role of endothelial shear stress in the natural history of coronary atherosclerosis and vascular remodeling. *Journal of the American College of Cardiology*.

[54] DeFronzo R. A. (2010). Insulin resistance, lipotoxicity, type 2 diabetes and atherosclerosis: the missing links. The claude bernard lecture 2009. *Diabetologia*.

[55] Lee K. K., Fortmann S. P., Fair J. M. (2009). Insulin resistance independently predicts the progression of coronary artery calcification. *American Heart Journal*.

[56] Beverly J. K., Budoff M. J. (2020). Atherosclerosis: pathophysiology of insulin resistance, hyperglycemia, hyperlipidemia, and inflammation. *Journal of Diabetes*.

[57] Montecucco F., Steffens S., Mach F. (2008). Insulin resistance: a proinflammatory state mediated by lipid-induced signaling dysfunction and involved in atherosclerotic plaque instability. *Mediators of Inflammation*.

[58] Biddinger S. B., Hernandez-Ono A., Rask-Madsen C. (2008). Hepatic insulin resistance is sufficient to produce dyslipidemia and susceptibility to atherosclerosis. *Cell Metabolism*.

