# Supplementary material for: Exploring the Therapeutic Mechanisms of Huzhang–Shanzha Herb Pair against Coronary Heart Disease by Network Pharmacology and Molecular Docking
Source: Evid Based Complement Alternat Med. 2021 Nov 30;2021:5569666. doi: 10.1155/2021/5569666 (PMC8651359; doi:10.1155/2021/5569666)

**Supplementary Table S1:** The information of all ingredients of HSHP for coronary heart disease.

| Drug | TCMID | Ingredient |
| --- | --- | --- |
| *P. cuspidatum* | TCMID:1367 | Anthraquinone |
| hawthorn | TCMID:23381 | Caffeic Acid Dimethyl Ether |
| *P. cuspidatum* | TCMID:3308 | (+)-Catechin |
| hawthorn | TCMID:3767 | Citronellal |
| hawthorn | TCMID:6329 | Dimethyl Camphorate |
| hawthorn | TCMID:25839 | 4-(1,5-Dimethyl-1,4-Hexadienyl)-1-Methyl-Cyclohexene |
| *P. cuspidatum* | TCMID:25838 | 3,5-Dimethyl-4-Methoxybenzoic Acid |
| hawthorn | TCMID:6317 | 1,2-Dimethylbenzene |
| hawthorn | TCMID:6328 | 3,5-Dimethylbutylbenzene |
| *P. cuspidatum* | TCMID:6775 | Emodin |
| *P. cuspidatum* | TCMID:6776 | Emodin Anthrone |
| hawthorn | TCMID:6853 | Epicatechin |
| hawthorn | TCMID:7280 | Eriodictyol-7,3-Diglucoside |
| hawthorn | TCMID:7461 | 1-Ethyl-2-Methylbenzene |
| hawthorn | TCMID:30462 | 1-Ethyl-4,8-Dimethoxy-Beta-Carboline |
| *P. cuspidatum* | TCMID:7713 | Fangchinoline |
| *P. cuspidatum* | TCMID:8094 | Gallicacid |
| hawthorn | TCMID:25961 | Gamma-Decanolactone |
| hawthorn | TCMID:9488 | 20-Hexadecanoylingenol |
| hawthorn | TCMID:12888 | Linoleyl Acetate |
| hawthorn | TCMID:14148 | Methylbenzene |
| hawthorn | TCMID:14263 | Methylcyclohexane |
| hawthorn | TCMID:14265 | 4-Methylcyclohexanone |
| hawthorn | TCMID:31632 | Methylheptenone |
| hawthorn | TCMID:14480 | 3-Methylhexane |
| hawthorn | TCMID:31636 | 3-Methylhistidin |
| hawthorn | TCMID:16283 | 12-Oxoarundoin |
| *P. cuspidatum* | TCMID:25027 | Polygalacic Acid |
| *P. cuspidatum* | TCMID:31893 | Quillaic Acid |
| *P. cuspidatum* | TCMID:18628 | Resveratrol |
| hawthorn | TCMID:20265 | Stearin |
| *P. cuspidatum* | TCMID:23118 | Trans-Resveratrol |
| hawthorn | TCMID:21949 | 3,4,4-Trimethyl-2-Hexene |
| hawthorn | TCMID:21926 | 1,2,3-Trimethylbenzene |
| hawthorn | TCMID:21938 | 3,7,11-Trimethyldodeca-1,7,10-Trien-3-Ol-9-One |
| hawthorn | TCMID:22254 | Ursolicacid |

**Supplementary Table S2:** Active ingredient parameters of HSHP.

| Parameter | Predicted candidate targets score | Enriched KEGG pathways/GO terms/TTD diseases/OMIM diseases adjusted P_value |
| --- | --- | --- |
| Cutoff | 20 | 0.05 |

**Supplementary Table S3:** All genes of HSHP in the treatment of CHD.

| Drug | Symbol |
| --- | --- |
| *P. cuspidatum* | ESR1 |
| *P. cuspidatum* | PTGS1 |
| *P. cuspidatum* | PTGS2 |
| *P. cuspidatum* | ACHE |
| *P. cuspidatum* | ADRA1A |
| *P. cuspidatum* | ADRA1B |
| *P. cuspidatum* | ADRA1D |
| *P. cuspidatum* | ADRA2A |
| *P. cuspidatum* | ADRA2B |
| *P. cuspidatum* | ADRA2C |
| *P. cuspidatum* | AKR1C1 |
| *P. cuspidatum* | ALOX5 |
| *P. cuspidatum* | ANXA1 |
| *P. cuspidatum* | APLP1 |
| *P. cuspidatum* | AR |
| *P. cuspidatum* | CALCA |
| *P. cuspidatum* | CHRNA7 |
| *P. cuspidatum* | DGKA |
| *P. cuspidatum* | ELOVL4 |
| *P. cuspidatum* | ESR2 |
| *P. cuspidatum* | ESRRG |
| *P. cuspidatum* | F12 |
| *P. cuspidatum* | FADS1 |
| *P. cuspidatum* | FADS2 |
| *P. cuspidatum* | INS |
| *P. cuspidatum* | NR1I2 |
| *P. cuspidatum* | NR3C1 |
| *P. cuspidatum* | PGR |
| *P. cuspidatum* | PHKG2 |
| *P. cuspidatum* | PPP2CA |
| *P. cuspidatum* | PPP2CB |
| *P. cuspidatum* | PRKCA |
| *P. cuspidatum* | PRKCB |
| *P. cuspidatum* | PTGER1 |
| *P. cuspidatum* | PTGER2 |
| *P. cuspidatum* | PTGER3 |
| *P. cuspidatum* | PTGER4 |
| *P. cuspidatum* | SEC14L2 |
| *P. cuspidatum* | SEC14L3 |
| *P. cuspidatum* | SEC14L4 |
| *P. cuspidatum* | SERPINB7 |
| *P. cuspidatum* | SLC8A1 |
| *P. cuspidatum* | SRD5A1 |
| *P. cuspidatum* | TRPV1 |
| *P. cuspidatum* | TYR |
| *P. cuspidatum* | BCHE |
| *P. cuspidatum* | CACNA1G |
| *P. cuspidatum* | CACNA1H |
| *P. cuspidatum* | CFTR |
| *P. cuspidatum* | CHRM2 |
| *P. cuspidatum* | CHRM3 |
| *P. cuspidatum* | CHRNA2 |
| *P. cuspidatum* | CNR1 |
| *P. cuspidatum* | CNR2 |
| *P. cuspidatum* | COL27A1 |
| *P. cuspidatum* | CSNK2A1 |
| *P. cuspidatum* | CYP11B1 |
| *P. cuspidatum* | CYP11B2 |
| *P. cuspidatum* | CYP17A1 |
| *P. cuspidatum* | DRD2 |
| *P. cuspidatum* | EDNRA |
| *P. cuspidatum* | HNRNPK |
| *P. cuspidatum* | HSPA5 |
| *P. cuspidatum* | HTR3A |
| *P. cuspidatum* | IKBKB |
| *P. cuspidatum* | KCNE2 |
| *P. cuspidatum* | LCT |
| *P. cuspidatum* | MTTP |
| *P. cuspidatum* | NFKB1 |
| *P. cuspidatum* | NFKB2 |
| *P. cuspidatum* | NFKBIA |
| *P. cuspidatum* | NQO2 |
| *P. cuspidatum* | NR3C2 |
| *P. cuspidatum* | OPRK1 |
| *P. cuspidatum* | PPARA |
| *P. cuspidatum* | PRKAA1 |
| *P. cuspidatum* | PRKAA2 |
| *P. cuspidatum* | PRKAB1 |
| *P. cuspidatum* | PRKAB2 |
| *P. cuspidatum* | PRKAG1 |
| *P. cuspidatum* | PRKAG2 |
| *P. cuspidatum* | PRKAG3 |
| *P. cuspidatum* | PRLR |
| *P. cuspidatum* | PTGIS |
| *P. cuspidatum* | RARA |
| *P. cuspidatum* | RARB |
| *P. cuspidatum* | RARG |
| *P. cuspidatum* | RPS6KA3 |
| *P. cuspidatum* | RXRA |
| *P. cuspidatum* | RXRB |
| *P. cuspidatum* | RXRG |
| *P. cuspidatum* | SIRT1 |
| *P. cuspidatum* | SLC18A2 |
| *P. cuspidatum* | SOAT |
| *P. cuspidatum* | SOAT2 |
| *P. cuspidatum* | TCAF1 |
| *P. cuspidatum* | TNF |
| *P. cuspidatum* | TP53 |
| *P. cuspidatum* | VKORC1 |
| *P. cuspidatum* | VKORC1L1 |
| hawthorn | AR |
| hawthorn | ADH1C |
| hawthorn | ADH1A |
| hawthorn | ADH1B |
| hawthorn | ALDH2 |
| hawthorn | CAT |
| hawthorn | DLG4 |
| hawthorn | GABRB3 |
| hawthorn | GAMT |
| hawthorn | GATM |
| hawthorn | GUCY1B3 |
| hawthorn | IYD |
| hawthorn | KCNA1 |
| hawthorn | KCNA10 |
| hawthorn | KCNA2 |
| hawthorn | KCNA3 |
| hawthorn | KCNA4 |
| hawthorn | KCNA5 |
| hawthorn | KCNA6 |
| hawthorn | KCNA7 |
| hawthorn | KCNB1 |
| hawthorn | KCNB2 |
| hawthorn | KCNC1 |
| hawthorn | KCNC2 |
| hawthorn | KCNC3 |
| hawthorn | KCND1 |
| hawthorn | KCND2 |
| hawthorn | KCND3 |
| hawthorn | KCNK4 |
| hawthorn | KCNQ1 |
| hawthorn | PRKAB1 |
| hawthorn | RNASE1 |
| hawthorn | TPO |
| hawthorn | CYP19A1 |
| hawthorn | ESR1 |
| hawthorn | F2 |
| hawthorn | GABRB2 |
| hawthorn | GPR27 |
| hawthorn | NR3C2 |
| hawthorn | OPRK1 |
| hawthorn | PGR |
| hawthorn | CYP17A1 |
| hawthorn | ELOVL4 |
| hawthorn | FADS1 |
| hawthorn | FADS2 |
| hawthorn | NR3C1 |
| hawthorn | PTGS1 |
| hawthorn | PTGS2 |
| hawthorn | SLC8A1 |
| hawthorn | TRPV1 |
| hawthorn | ANXA1 |
| hawthorn | F12 |
| hawthorn | MTTP |
| hawthorn | NPR1 |
| hawthorn | PDE10A |
| hawthorn | PDE4B |
| hawthorn | PDE4D |
| hawthorn | PRKCA |
| hawthorn | PRLR |
| hawthorn | PTGER2 |
| hawthorn | PTGER3 |
| hawthorn | PTGER4 |
| hawthorn | SOAT2 |
| hawthorn | SRD5A1 |
| hawthorn | ACHE |
| hawthorn | ACSL3 |
| hawthorn | ACSL4 |
| hawthorn | ADA |
| hawthorn | ADORA1 |
| hawthorn | ADORA2A |
| hawthorn | ADORA2B |
| hawthorn | ADRA1A |
| hawthorn | ADRA1B |
| hawthorn | ADRA1D |
| hawthorn | ADRA2A |
| hawthorn | ADRA2B |
| hawthorn | ADRA2C |
| hawthorn | ADRB1 |
| hawthorn | ADRB2 |
| hawthorn | ADRB3 |
| hawthorn | AKR1C2 |
| hawthorn | AKR1D1 |
| hawthorn | ALOX5 |
| hawthorn | AMPD3 |
| hawthorn | ATM |
| hawthorn | ATP1A1 |
| hawthorn | BCHE |
| hawthorn | CARTPT |
| hawthorn | CES1 |
| hawthorn | CHRM1 |
| hawthorn | CHRM2 |
| hawthorn | CHRM3 |
| hawthorn | CHRNA10 |
| hawthorn | CNR1 |
| hawthorn | CNR2 |
| hawthorn | COL27A1 |
| hawthorn | COLQ |
| hawthorn | COX1 |
| hawthorn | COX2 |
| hawthorn | COX3 |
| hawthorn | COX4I1 |
| hawthorn | COX5A |
| hawthorn | COX5B |
| hawthorn | COX6A2 |
| hawthorn | COX6B1 |
| hawthorn | COX6C |
| hawthorn | COX7A1 |
| hawthorn | COX7B |
| hawthorn | COX7C |
| hawthorn | COX8A |
| hawthorn | CX3CR1 |
| hawthorn | DGKA |
| hawthorn | DRD1 |
| hawthorn | DRD2 |
| hawthorn | ESRRG |
| hawthorn | FABP6 |
| hawthorn | FECH |
| hawthorn | FFAR1 |
| hawthorn | GLRA3 |
| hawthorn | HAL |
| hawthorn | HAP1 |
| hawthorn | HARS |
| hawthorn | HARS2 |
| hawthorn | HDAC2 |
| hawthorn | HDC |
| hawthorn | IDNK |
| hawthorn | ITPR1 |
| hawthorn | ITPR2 |
| hawthorn | ITPR3 |
| hawthorn | MAOA |
| hawthorn | MAOB |
| hawthorn | NR1H4 |
| hawthorn | NR1I2 |
| hawthorn | NT5E |
| hawthorn | OAT1 |
| hawthorn | PCK1 |
| hawthorn | PDE11A |
| hawthorn | PDE1A |
| hawthorn | PDE1B |
| hawthorn | PDE1C |
| hawthorn | PDE2A |
| hawthorn | PDE3A |
| hawthorn | PDE3B |
| hawthorn | PDE4A |
| hawthorn | PDE4C |
| hawthorn | PDE5A |
| hawthorn | PDE6A |
| hawthorn | PDE6B |
| hawthorn | PDE6C |
| hawthorn | PDE7A |
| hawthorn | PDE7B |
| hawthorn | PDE8A |
| hawthorn | PDE8B |
| hawthorn | PDE9A |
| hawthorn | PGD |
| hawthorn | PIK3CA |
| hawthorn | PIK3CB |
| hawthorn | PIK3CD |
| hawthorn | PIK3R1 |
| hawthorn | PLA2G1B |
| hawthorn | POLA2 |
| hawthorn | PPARD |
| hawthorn | PPARG |
| hawthorn | PPP2CA |
| hawthorn | PPP2CB |
| hawthorn | PRG3 |
| hawthorn | PRKCB |
| hawthorn | PRKCD |
| hawthorn | PRKDC |
| hawthorn | PTGER1 |
| hawthorn | RARA |
| hawthorn | RARB |
| hawthorn | RARG |
| hawthorn | RBP1 |
| hawthorn | RINT1 |
| hawthorn | RIPK1 |
| hawthorn | RXRA |
| hawthorn | RXRB |
| hawthorn | RXRG |
| hawthorn | RYR1 |
| hawthorn | S1PR5 |
| hawthorn | SEC14L2 |
| hawthorn | SEC14L3 |
| hawthorn | SEC14L4 |
| hawthorn | SLC18A2 |
| hawthorn | SLC38A3 |
| hawthorn | SLC38A7 |
| hawthorn | SLC6A2 |
| hawthorn | SLC6A3 |
| hawthorn | SLC6A4 |
| hawthorn | SOAT1 |
| hawthorn | SRD5A2 |
| hawthorn | TAAR1 |
| hawthorn | TACR2 |
| hawthorn | TNF |
| hawthorn | TRPA1 |
| hawthorn | TRPM8 |
| hawthorn | TRPV3 |
| hawthorn | TYR |
| hawthorn | WARS |
| hawthorn | WARS2 |
| hawthorn | WNT4 |

**Supplementary Table S4:** Compound-target pair information.

| Drug | TCMId | MolName | Symbol |
| --- | --- | --- | --- |
| *P. cuspidatum* | TCMID:7713 | Fangchinoline | ACHE |
| *P. cuspidatum* | TCMID:17239 | Physovenine | ACHE |
| hawthorn | TCMID:3767 | Citronellal | ACHE |
| hawthorn | TCMID:21938 | 3,7,11-Trimethyldodeca-1,7,10-Trien-3-Ol-9-One | ACSL3 |
| hawthorn | TCMID:21938 | 3,7,11-Trimethyldodeca-1,7,10-Trien-3-Ol-9-One | ACSL4 |
| hawthorn | TCMID:31632 | Methylheptenone | ADA |
| hawthorn | TCMID:21949 | 3,4,4-Trimethyl-2-Hexene | ADH1A |
| hawthorn | TCMID:14480 | 3-Methylhexane | ADH1A |
| hawthorn | TCMID:6317 | 1,2-Dimethylbenzene | ADH1A |
| hawthorn | TCMID:14263 | Methylcyclohexane | ADH1A |
| hawthorn | TCMID:25839 | 4-(1,5-Dimethyl-1,4-Hexadienyl)-1-Methyl-Cyclohexene | ADH1A |
| hawthorn | TCMID:21949 | 3,4,4-Trimethyl-2-Hexene | ADH1B |
| hawthorn | TCMID:14480 | 3-Methylhexane | ADH1B |
| hawthorn | TCMID:6317 | 1,2-Dimethylbenzene | ADH1B |
| hawthorn | TCMID:14263 | Methylcyclohexane | ADH1B |
| hawthorn | TCMID:25839 | 4-(1,5-Dimethyl-1,4-Hexadienyl)-1-Methyl-Cyclohexene | ADH1B |
| hawthorn | TCMID:21949 | 3,4,4-Trimethyl-2-Hexene | ADH1C |
| hawthorn | TCMID:14480 | 3-Methylhexane | ADH1C |
| hawthorn | TCMID:6317 | 1,2-Dimethylbenzene | ADH1C |
| hawthorn | TCMID:14263 | Methylcyclohexane | ADH1C |
| hawthorn | TCMID:25839 | 4-(1,5-Dimethyl-1,4-Hexadienyl)-1-Methyl-Cyclohexene | ADH1C |
| hawthorn | TCMID:6329 | Dimethyl Camphorate | ADH1C |
| hawthorn | TCMID:31632 | Methylheptenone | ADORA1 |
| hawthorn | TCMID:31632 | Methylheptenone | ADORA2A |
| hawthorn | TCMID:31632 | Methylheptenone | ADORA2B |
| *P. cuspidatum* | TCMID:23118 | Trans-Resveratrol | ADRA1A |
| *P. cuspidatum* | TCMID:18628 | Resveratrol | ADRA1A |
| hawthorn | TCMID:6328 | 3,5-Dimethylbutylbenzene | ADRA1A |
| *P. cuspidatum* | TCMID:23118 | Trans-Resveratrol | ADRA1B |
| *P. cuspidatum* | TCMID:18628 | Resveratrol | ADRA1B |
| hawthorn | TCMID:6328 | 3,5-Dimethylbutylbenzene | ADRA1B |
| *P. cuspidatum* | TCMID:23118 | Trans-Resveratrol | ADRA1D |
| *P. cuspidatum* | TCMID:18628 | Resveratrol | ADRA1D |
| hawthorn | TCMID:6328 | 3,5-Dimethylbutylbenzene | ADRA1D |
| *P. cuspidatum* | TCMID:23118 | Trans-Resveratrol | ADRA2A |
| *P. cuspidatum* | TCMID:18628 | Resveratrol | ADRA2A |
| hawthorn | TCMID:6328 | 3,5-Dimethylbutylbenzene | ADRA2A |
| *P. cuspidatum* | TCMID:23118 | Trans-Resveratrol | ADRA2B |
| *P. cuspidatum* | TCMID:18628 | Resveratrol | ADRA2B |
| hawthorn | TCMID:6328 | 3,5-Dimethylbutylbenzene | ADRA2B |
| *P. cuspidatum* | TCMID:23118 | Trans-Resveratrol | ADRA2C |
| *P. cuspidatum* | TCMID:18628 | Resveratrol | ADRA2C |
| hawthorn | TCMID:6328 | 3,5-Dimethylbutylbenzene | ADRA2C |
| hawthorn | TCMID:6328 | 3,5-Dimethylbutylbenzene | ADRB1 |
| hawthorn | TCMID:6328 | 3,5-Dimethylbutylbenzene | ADRB2 |
| hawthorn | TCMID:6328 | 3,5-Dimethylbutylbenzene | ADRB3 |
| *P. cuspidatum* | TCMID:25838 | 3,5-Dimethyl-4-Methoxybenzoic Acid | AKR1C1 |
| *P. cuspidatum* | TCMID:8094 | Gallicacid | AKR1C1 |
| hawthorn | TCMID:6329 | Dimethyl Camphorate | AKR1C2 |
| hawthorn | TCMID:6329 | Dimethyl Camphorate | AKR1D1 |
| hawthorn | TCMID:21949 | 3,4,4-Trimethyl-2-Hexene | ALDH2 |
| hawthorn | TCMID:14480 | 3-Methylhexane | ALDH2 |
| hawthorn | TCMID:6317 | 1,2-Dimethylbenzene | ALDH2 |
| hawthorn | TCMID:14263 | Methylcyclohexane | ALDH2 |
| hawthorn | TCMID:25839 | 4-(1,5-Dimethyl-1,4-Hexadienyl)-1-Methyl-Cyclohexene | ALDH2 |
| *P. cuspidatum* | TCMID:3308 | (+)-Catechin | ALOX5 |
| *P. cuspidatum* | TCMID:8094 | Gallicacid | ALOX5 |
| hawthorn | TCMID:6853 | Epicatechin | ALOX5 |
| hawthorn | TCMID:31632 | Methylheptenone | AMPD3 |
| *P. cuspidatum* | TCMID:31893 | Quillaic Acid | ANXA1 |
| *P. cuspidatum* | TCMID:25027 | Polygalacic Acid | ANXA1 |
| hawthorn | TCMID:16283 | 12-Oxoarundoin | ANXA1 |
| hawthorn | TCMID:22254 | Ursolicacid | ANXA1 |
| *P. cuspidatum* | TCMID:23118 | Trans-Resveratrol | APLP1 |
| *P. cuspidatum* | TCMID:18628 | Resveratrol | APLP1 |
| *P. cuspidatum* | TCMID:31893 | Quillaic Acid | AR |
| *P. cuspidatum* | TCMID:25027 | Polygalacic Acid | AR |
| hawthorn | TCMID:20265 | Stearin | AR |
| hawthorn | TCMID:12888 | Linoleyl Acetate | AR |
| hawthorn | TCMID:14265 | 4-Methylcyclohexanone | AR |
| hawthorn | TCMID:21938 | 3,7,11-Trimethyldodeca-1,7,10-Trien-3-Ol-9-One | AR |
| hawthorn | TCMID:16283 | 12-Oxoarundoin | AR |
| hawthorn | TCMID:25961 | Gamma-Decanolactone | AR |
| hawthorn | TCMID:22254 | Ursolicacid | AR |
| hawthorn | TCMID:6329 | Dimethyl Camphorate | AR |
| hawthorn | TCMID:31632 | Methylheptenone | ATM |
| hawthorn | TCMID:19075 | Ruvoside | ATP1A1 |
| *P. cuspidatum* | TCMID:7713 | Fangchinoline | BCHE |
| hawthorn | TCMID:3767 | Citronellal | BCHE |
| *P. cuspidatum* | TCMID:1367 | Anthraquinone | CACNA1G |
| *P. cuspidatum* | TCMID:1367 | Anthraquinone | CACNA1H |
| *P. cuspidatum* | TCMID:23118 | Trans-Resveratrol | CALCA |
| *P. cuspidatum* | TCMID:18628 | Resveratrol | CALCA |
| hawthorn | TCMID:6328 | 3,5-Dimethylbutylbenzene | CARTPT |
| hawthorn | TCMID:21949 | 3,4,4-Trimethyl-2-Hexene | CAT |
| hawthorn | TCMID:14480 | 3-Methylhexane | CAT |
| hawthorn | TCMID:6317 | 1,2-Dimethylbenzene | CAT |
| hawthorn | TCMID:14263 | Methylcyclohexane | CAT |
| hawthorn | TCMID:25839 | 4-(1,5-Dimethyl-1,4-Hexadienyl)-1-Methyl-Cyclohexene | CAT |
| hawthorn | TCMID:6329 | Dimethyl Camphorate | CES1 |
| *P. cuspidatum* | TCMID:3615 | Chrysophanol | CFTR |
| hawthorn | TCMID:351 | Acetylcholine | CHRM1 |
| *P. cuspidatum* | TCMID:7713 | Fangchinoline | CHRM2 |
| hawthorn | TCMID:351 | Acetylcholine | CHRM2 |
| *P. cuspidatum* | TCMID:7713 | Fangchinoline | CHRM3 |
| hawthorn | TCMID:351 | Acetylcholine | CHRM3 |
| hawthorn | TCMID:351 | Acetylcholine | CHRNA10 |
| *P. cuspidatum* | TCMID:7713 | Fangchinoline | CHRNA2 |
| *P. cuspidatum* | TCMID:23118 | Trans-Resveratrol | CHRNA7 |
| *P. cuspidatum* | TCMID:18628 | Resveratrol | CHRNA7 |
| *P. cuspidatum* | TCMID:3308 | (+)-Catechin | CNR1 |
| hawthorn | TCMID:6853 | Epicatechin | CNR1 |
| *P. cuspidatum* | TCMID:3308 | (+)-Catechin | CNR2 |
| hawthorn | TCMID:6853 | Epicatechin | CNR2 |
| *P. cuspidatum* | TCMID:25838 | 3,5-Dimethyl-4-Methoxybenzoic Acid | COL27A1 |
| hawthorn | TCMID:23381 | Caffeic Acid Dimethyl Ether | COL27A1 |
| hawthorn | TCMID:3767 | Citronellal | COLQ |
| hawthorn | TCMID:6329 | Dimethyl Camphorate | COX1 |
| hawthorn | TCMID:6329 | Dimethyl Camphorate | COX2 |
| hawthorn | TCMID:6329 | Dimethyl Camphorate | COX3 |
| hawthorn | TCMID:6329 | Dimethyl Camphorate | COX4I1 |
| hawthorn | TCMID:6329 | Dimethyl Camphorate | COX5A |
| hawthorn | TCMID:6329 | Dimethyl Camphorate | COX5B |
| hawthorn | TCMID:6329 | Dimethyl Camphorate | COX6A2 |
| hawthorn | TCMID:6329 | Dimethyl Camphorate | COX6B1 |
| hawthorn | TCMID:6329 | Dimethyl Camphorate | COX6C |
| hawthorn | TCMID:6329 | Dimethyl Camphorate | COX7A1 |
| hawthorn | TCMID:6329 | Dimethyl Camphorate | COX7B |
| hawthorn | TCMID:6329 | Dimethyl Camphorate | COX7C |
| hawthorn | TCMID:6329 | Dimethyl Camphorate | COX8A |
| *P. cuspidatum* | TCMID:23118 | Trans-Resveratrol | CSNK2A1 |
| hawthorn | TCMID:31632 | Methylheptenone | CX3CR1 |
| *P. cuspidatum* | TCMID:1367 | Anthraquinone | CYP11B1 |
| *P. cuspidatum* | TCMID:1367 | Anthraquinone | CYP11B2 |
| *P. cuspidatum* | TCMID:31893 | Quillaic Acid | CYP17A1 |
| hawthorn | TCMID:31632 | Methylheptenone | CYP17A1 |
| hawthorn | TCMID:16283 | 12-Oxoarundoin | CYP17A1 |
| hawthorn | TCMID:22254 | Ursolicacid | CYP17A1 |
| hawthorn | TCMID:12888 | Linoleyl Acetate | CYP19A1 |
| hawthorn | TCMID:31632 | Methylheptenone | CYP19A1 |
| hawthorn | TCMID:16283 | 12-Oxoarundoin | CYP19A1 |
| hawthorn | TCMID:16283 | 12-Oxoarundoin | CYP19A1 |
| *P. cuspidatum* | TCMID:3308 | (+)-Catechin | DGKA |
| *P. cuspidatum* | TCMID:8094 | Gallicacid | DGKA |
| hawthorn | TCMID:6853 | Epicatechin | DGKA |
| hawthorn | TCMID:21949 | 3,4,4-Trimethyl-2-Hexene | DLG4 |
| hawthorn | TCMID:14480 | 3-Methylhexane | DLG4 |
| hawthorn | TCMID:6317 | 1,2-Dimethylbenzene | DLG4 |
| hawthorn | TCMID:14263 | Methylcyclohexane | DLG4 |
| hawthorn | TCMID:25839 | 4-(1,5-Dimethyl-1,4-Hexadienyl)-1-Methyl-Cyclohexene | DLG4 |
| hawthorn | TCMID:6328 | 3,5-Dimethylbutylbenzene | DRD1 |
| *P. cuspidatum* | TCMID:7713 | Fangchinoline | DRD2 |
| hawthorn | TCMID:6328 | 3,5-Dimethylbutylbenzene | DRD2 |
| *P. cuspidatum* | TCMID:25838 | 3,5-Dimethyl-4-Methoxybenzoic Acid | EDNRA |
| *P. cuspidatum* | TCMID:31893 | Quillaic Acid | ELOVL4 |
| *P. cuspidatum* | TCMID:25027 | Polygalacic Acid | ELOVL4 |
| hawthorn | TCMID:12888 | Linoleyl Acetate | ELOVL4 |
| hawthorn | TCMID:21938 | 3,7,11-Trimethyldodeca-1,7,10-Trien-3-Ol-9-One | ELOVL4 |
| hawthorn | TCMID:22254 | Ursolicacid | ELOVL4 |
| *P. cuspidatum* | TCMID:31893 | Quillaic Acid | ESR1 |
| *P. cuspidatum* | TCMID:23118 | Trans-Resveratrol | ESR1 |
| *P. cuspidatum* | TCMID:25027 | Polygalacic Acid | ESR1 |
| *P. cuspidatum* | TCMID:6775 | Emodin | ESR1 |
| *P. cuspidatum* | TCMID:18628 | Resveratrol | ESR1 |
| *P. cuspidatum* | TCMID:6776 | Emodin Anthrone | ESR1 |
| hawthorn | TCMID:12888 | Linoleyl Acetate | ESR1 |
| hawthorn | TCMID:31632 | Methylheptenone | ESR1 |
| hawthorn | TCMID:16283 | 12-Oxoarundoin | ESR1 |
| hawthorn | TCMID:22254 | Ursolicacid | ESR1 |
| *P. cuspidatum* | TCMID:23118 | Trans-Resveratrol | ESR2 |
| *P. cuspidatum* | TCMID:18628 | Resveratrol | ESR2 |
| *P. cuspidatum* | TCMID:23118 | Trans-Resveratrol | ESRRG |
| *P. cuspidatum* | TCMID:18628 | Resveratrol | ESRRG |
| hawthorn | TCMID:6329 | Dimethyl Camphorate | ESRRG |
| *P. cuspidatum* | TCMID:31893 | Quillaic Acid | F12 |
| *P. cuspidatum* | TCMID:25027 | Polygalacic Acid | F12 |
| hawthorn | TCMID:3767 | Citronellal | F12 |
| hawthorn | TCMID:22254 | Ursolicacid | F12 |
| hawthorn | TCMID:6328 | 3,5-Dimethylbutylbenzene | F2 |
| hawthorn | TCMID:7461 | 1-Ethyl-2-Methylbenzene | F2 |
| hawthorn | TCMID:14148 | Methylbenzene | F2 |
| hawthorn | TCMID:21926 | 1,2,3-Trimethylbenzene | F2 |
| hawthorn | TCMID:6329 | Dimethyl Camphorate | FABP6 |
| *P. cuspidatum* | TCMID:31893 | Quillaic Acid | FADS1 |
| *P. cuspidatum* | TCMID:25027 | Polygalacic Acid | FADS1 |
| hawthorn | TCMID:12888 | Linoleyl Acetate | FADS1 |
| hawthorn | TCMID:21938 | 3,7,11-Trimethyldodeca-1,7,10-Trien-3-Ol-9-One | FADS1 |
| hawthorn | TCMID:22254 | Ursolicacid | FADS1 |
| *P. cuspidatum* | TCMID:31893 | Quillaic Acid | FADS2 |
| *P. cuspidatum* | TCMID:25027 | Polygalacic Acid | FADS2 |
| hawthorn | TCMID:12888 | Linoleyl Acetate | FADS2 |
| hawthorn | TCMID:21938 | 3,7,11-Trimethyldodeca-1,7,10-Trien-3-Ol-9-One | FADS2 |
| hawthorn | TCMID:22254 | Ursolicacid | FADS2 |
| hawthorn | TCMID:6329 | Dimethyl Camphorate | FECH |
| hawthorn | TCMID:21938 | 3,7,11-Trimethyldodeca-1,7,10-Trien-3-Ol-9-One | FFAR1 |
| hawthorn | TCMID:6328 | 3,5-Dimethylbutylbenzene | GABRB2 |
| hawthorn | TCMID:7461 | 1-Ethyl-2-Methylbenzene | GABRB2 |
| hawthorn | TCMID:14148 | Methylbenzene | GABRB2 |
| hawthorn | TCMID:21926 | 1,2,3-Trimethylbenzene | GABRB2 |
| hawthorn | TCMID:19075 | Ruvoside | GABRB3 |
| hawthorn | TCMID:6328 | 3,5-Dimethylbutylbenzene | GABRB3 |
| hawthorn | TCMID:7461 | 1-Ethyl-2-Methylbenzene | GABRB3 |
| hawthorn | TCMID:14148 | Methylbenzene | GABRB3 |
| hawthorn | TCMID:21926 | 1,2,3-Trimethylbenzene | GABRB3 |
| hawthorn | TCMID:21949 | 3,4,4-Trimethyl-2-Hexene | GAMT |
| hawthorn | TCMID:14480 | 3-Methylhexane | GAMT |
| hawthorn | TCMID:6317 | 1,2-Dimethylbenzene | GAMT |
| hawthorn | TCMID:14263 | Methylcyclohexane | GAMT |
| hawthorn | TCMID:25839 | 4-(1,5-Dimethyl-1,4-Hexadienyl)-1-Methyl-Cyclohexene | GAMT |
| hawthorn | TCMID:21949 | 3,4,4-Trimethyl-2-Hexene | GATM |
| hawthorn | TCMID:14480 | 3-Methylhexane | GATM |
| hawthorn | TCMID:6317 | 1,2-Dimethylbenzene | GATM |
| hawthorn | TCMID:14263 | Methylcyclohexane | GATM |
| hawthorn | TCMID:25839 | 4-(1,5-Dimethyl-1,4-Hexadienyl)-1-Methyl-Cyclohexene | GATM |
| hawthorn | TCMID:19075 | Ruvoside | GLRA3 |
| hawthorn | TCMID:6328 | 3,5-Dimethylbutylbenzene | GPR27 |
| hawthorn | TCMID:7461 | 1-Ethyl-2-Methylbenzene | GPR27 |
| hawthorn | TCMID:14148 | Methylbenzene | GPR27 |
| hawthorn | TCMID:21926 | 1,2,3-Trimethylbenzene | GPR27 |
| hawthorn | TCMID:21949 | 3,4,4-Trimethyl-2-Hexene | GUCY1B3 |
| hawthorn | TCMID:14480 | 3-Methylhexane | GUCY1B3 |
| hawthorn | TCMID:6317 | 1,2-Dimethylbenzene | GUCY1B3 |
| hawthorn | TCMID:14263 | Methylcyclohexane | GUCY1B3 |
| hawthorn | TCMID:25839 | 4-(1,5-Dimethyl-1,4-Hexadienyl)-1-Methyl-Cyclohexene | GUCY1B3 |
| hawthorn | TCMID:31636 | 3-Methylhistidin | HAL |
| hawthorn | TCMID:31632 | Methylheptenone | HAP1 |
| hawthorn | TCMID:31636 | 3-Methylhistidin | HARS |
| hawthorn | TCMID:31636 | 3-Methylhistidin | HARS2 |
| hawthorn | TCMID:31632 | Methylheptenone | HDAC2 |
| hawthorn | TCMID:31636 | 3-Methylhistidin | HDC |
| *P. cuspidatum* | TCMID:25838 | 3,5-Dimethyl-4-Methoxybenzoic Acid | HNRNPK |
| *P. cuspidatum* | TCMID:25838 | 3,5-Dimethyl-4-Methoxybenzoic Acid | HSPA5 |
| *P. cuspidatum* | TCMID:7713 | Fangchinoline | HTR3A |
| hawthorn | TCMID:31632 | Methylheptenone | IDNK |
| *P. cuspidatum* | TCMID:25838 | 3,5-Dimethyl-4-Methoxybenzoic Acid | IKBKB |
| *P. cuspidatum* | TCMID:23118 | Trans-Resveratrol | INS |
| *P. cuspidatum* | TCMID:18628 | Resveratrol | INS |
| hawthorn | TCMID:31632 | Methylheptenone | ITPR1 |
| hawthorn | TCMID:31632 | Methylheptenone | ITPR2 |
| hawthorn | TCMID:31632 | Methylheptenone | ITPR3 |
| hawthorn | TCMID:21949 | 3,4,4-Trimethyl-2-Hexene | IYD |
| hawthorn | TCMID:14480 | 3-Methylhexane | IYD |
| hawthorn | TCMID:6317 | 1,2-Dimethylbenzene | IYD |
| hawthorn | TCMID:14263 | Methylcyclohexane | IYD |
| hawthorn | TCMID:25839 | 4-(1,5-Dimethyl-1,4-Hexadienyl)-1-Methyl-Cyclohexene | IYD |
| hawthorn | TCMID:21949 | 3,4,4-Trimethyl-2-Hexene | KCNA1 |
| hawthorn | TCMID:14480 | 3-Methylhexane | KCNA1 |
| hawthorn | TCMID:6317 | 1,2-Dimethylbenzene | KCNA1 |
| hawthorn | TCMID:14263 | Methylcyclohexane | KCNA1 |
| hawthorn | TCMID:25839 | 4-(1,5-Dimethyl-1,4-Hexadienyl)-1-Methyl-Cyclohexene | KCNA1 |
| hawthorn | TCMID:21949 | 3,4,4-Trimethyl-2-Hexene | KCNA10 |
| hawthorn | TCMID:14480 | 3-Methylhexane | KCNA10 |
| hawthorn | TCMID:6317 | 1,2-Dimethylbenzene | KCNA10 |
| hawthorn | TCMID:14263 | Methylcyclohexane | KCNA10 |
| hawthorn | TCMID:25839 | 4-(1,5-Dimethyl-1,4-Hexadienyl)-1-Methyl-Cyclohexene | KCNA10 |
| hawthorn | TCMID:21949 | 3,4,4-Trimethyl-2-Hexene | KCNA2 |
| hawthorn | TCMID:14480 | 3-Methylhexane | KCNA2 |
| hawthorn | TCMID:6317 | 1,2-Dimethylbenzene | KCNA2 |
| hawthorn | TCMID:14263 | Methylcyclohexane | KCNA2 |
| hawthorn | TCMID:25839 | 4-(1,5-Dimethyl-1,4-Hexadienyl)-1-Methyl-Cyclohexene | KCNA2 |
| hawthorn | TCMID:21949 | 3,4,4-Trimethyl-2-Hexene | KCNA3 |
| hawthorn | TCMID:14480 | 3-Methylhexane | KCNA3 |
| hawthorn | TCMID:6317 | 1,2-Dimethylbenzene | KCNA3 |
| hawthorn | TCMID:14263 | Methylcyclohexane | KCNA3 |
| hawthorn | TCMID:25839 | 4-(1,5-Dimethyl-1,4-Hexadienyl)-1-Methyl-Cyclohexene | KCNA3 |
| hawthorn | TCMID:21949 | 3,4,4-Trimethyl-2-Hexene | KCNA4 |
| hawthorn | TCMID:14480 | 3-Methylhexane | KCNA4 |
| hawthorn | TCMID:6317 | 1,2-Dimethylbenzene | KCNA4 |
| hawthorn | TCMID:14263 | Methylcyclohexane | KCNA4 |
| hawthorn | TCMID:25839 | 4-(1,5-Dimethyl-1,4-Hexadienyl)-1-Methyl-Cyclohexene | KCNA4 |
| hawthorn | TCMID:21949 | 3,4,4-Trimethyl-2-Hexene | KCNA5 |
| hawthorn | TCMID:14480 | 3-Methylhexane | KCNA5 |
| hawthorn | TCMID:6317 | 1,2-Dimethylbenzene | KCNA5 |
| hawthorn | TCMID:14263 | Methylcyclohexane | KCNA5 |
| hawthorn | TCMID:25839 | 4-(1,5-Dimethyl-1,4-Hexadienyl)-1-Methyl-Cyclohexene | KCNA5 |
| hawthorn | TCMID:21949 | 3,4,4-Trimethyl-2-Hexene | KCNA6 |
| hawthorn | TCMID:14480 | 3-Methylhexane | KCNA6 |
| hawthorn | TCMID:6317 | 1,2-Dimethylbenzene | KCNA6 |
| hawthorn | TCMID:14263 | Methylcyclohexane | KCNA6 |
| hawthorn | TCMID:25839 | 4-(1,5-Dimethyl-1,4-Hexadienyl)-1-Methyl-Cyclohexene | KCNA6 |
| hawthorn | TCMID:21949 | 3,4,4-Trimethyl-2-Hexene | KCNA7 |
| hawthorn | TCMID:14480 | 3-Methylhexane | KCNA7 |
| hawthorn | TCMID:6317 | 1,2-Dimethylbenzene | KCNA7 |
| hawthorn | TCMID:14263 | Methylcyclohexane | KCNA7 |
| hawthorn | TCMID:25839 | 4-(1,5-Dimethyl-1,4-Hexadienyl)-1-Methyl-Cyclohexene | KCNA7 |
| hawthorn | TCMID:21949 | 3,4,4-Trimethyl-2-Hexene | KCNB1 |
| hawthorn | TCMID:14480 | 3-Methylhexane | KCNB1 |
| hawthorn | TCMID:6317 | 1,2-Dimethylbenzene | KCNB1 |
| hawthorn | TCMID:14263 | Methylcyclohexane | KCNB1 |
| hawthorn | TCMID:25839 | 4-(1,5-Dimethyl-1,4-Hexadienyl)-1-Methyl-Cyclohexene | KCNB1 |
| hawthorn | TCMID:21949 | 3,4,4-Trimethyl-2-Hexene | KCNB2 |
| hawthorn | TCMID:14480 | 3-Methylhexane | KCNB2 |
| hawthorn | TCMID:6317 | 1,2-Dimethylbenzene | KCNB2 |
| hawthorn | TCMID:14263 | Methylcyclohexane | KCNB2 |
| hawthorn | TCMID:25839 | 4-(1,5-Dimethyl-1,4-Hexadienyl)-1-Methyl-Cyclohexene | KCNB2 |
| hawthorn | TCMID:21949 | 3,4,4-Trimethyl-2-Hexene | KCNC1 |
| hawthorn | TCMID:14480 | 3-Methylhexane | KCNC1 |
| hawthorn | TCMID:6317 | 1,2-Dimethylbenzene | KCNC1 |
| hawthorn | TCMID:14263 | Methylcyclohexane | KCNC1 |
| hawthorn | TCMID:25839 | 4-(1,5-Dimethyl-1,4-Hexadienyl)-1-Methyl-Cyclohexene | KCNC1 |
| hawthorn | TCMID:21949 | 3,4,4-Trimethyl-2-Hexene | KCNC2 |
| hawthorn | TCMID:14480 | 3-Methylhexane | KCNC2 |
| hawthorn | TCMID:6317 | 1,2-Dimethylbenzene | KCNC2 |
| hawthorn | TCMID:14263 | Methylcyclohexane | KCNC2 |
| hawthorn | TCMID:25839 | 4-(1,5-Dimethyl-1,4-Hexadienyl)-1-Methyl-Cyclohexene | KCNC2 |
| hawthorn | TCMID:21949 | 3,4,4-Trimethyl-2-Hexene | KCNC3 |
| hawthorn | TCMID:14480 | 3-Methylhexane | KCNC3 |
| hawthorn | TCMID:6317 | 1,2-Dimethylbenzene | KCNC3 |
| hawthorn | TCMID:14263 | Methylcyclohexane | KCNC3 |
| hawthorn | TCMID:25839 | 4-(1,5-Dimethyl-1,4-Hexadienyl)-1-Methyl-Cyclohexene | KCNC3 |
| hawthorn | TCMID:21949 | 3,4,4-Trimethyl-2-Hexene | KCND1 |
| hawthorn | TCMID:14480 | 3-Methylhexane | KCND1 |
| hawthorn | TCMID:6317 | 1,2-Dimethylbenzene | KCND1 |
| hawthorn | TCMID:14263 | Methylcyclohexane | KCND1 |
| hawthorn | TCMID:25839 | 4-(1,5-Dimethyl-1,4-Hexadienyl)-1-Methyl-Cyclohexene | KCND1 |
| hawthorn | TCMID:21949 | 3,4,4-Trimethyl-2-Hexene | KCND2 |
| hawthorn | TCMID:14480 | 3-Methylhexane | KCND2 |
| hawthorn | TCMID:6317 | 1,2-Dimethylbenzene | KCND2 |
| hawthorn | TCMID:14263 | Methylcyclohexane | KCND2 |
| hawthorn | TCMID:25839 | 4-(1,5-Dimethyl-1,4-Hexadienyl)-1-Methyl-Cyclohexene | KCND2 |
| hawthorn | TCMID:21949 | 3,4,4-Trimethyl-2-Hexene | KCND3 |
| hawthorn | TCMID:14480 | 3-Methylhexane | KCND3 |
| hawthorn | TCMID:6317 | 1,2-Dimethylbenzene | KCND3 |
| hawthorn | TCMID:14263 | Methylcyclohexane | KCND3 |
| hawthorn | TCMID:25839 | 4-(1,5-Dimethyl-1,4-Hexadienyl)-1-Methyl-Cyclohexene | KCND3 |
| *P. cuspidatum* | TCMID:3615 | Chrysophanol | KCNE2 |
| hawthorn | TCMID:21949 | 3,4,4-Trimethyl-2-Hexene | KCNK4 |
| hawthorn | TCMID:14480 | 3-Methylhexane | KCNK4 |
| hawthorn | TCMID:6317 | 1,2-Dimethylbenzene | KCNK4 |
| hawthorn | TCMID:14263 | Methylcyclohexane | KCNK4 |
| hawthorn | TCMID:25839 | 4-(1,5-Dimethyl-1,4-Hexadienyl)-1-Methyl-Cyclohexene | KCNK4 |
| hawthorn | TCMID:21949 | 3,4,4-Trimethyl-2-Hexene | KCNQ1 |
| hawthorn | TCMID:14480 | 3-Methylhexane | KCNQ1 |
| hawthorn | TCMID:6317 | 1,2-Dimethylbenzene | KCNQ1 |
| hawthorn | TCMID:14263 | Methylcyclohexane | KCNQ1 |
| hawthorn | TCMID:25839 | 4-(1,5-Dimethyl-1,4-Hexadienyl)-1-Methyl-Cyclohexene | KCNQ1 |
| *P. cuspidatum* | TCMID:1367 | Anthraquinone | LCT |
| hawthorn | TCMID:6328 | 3,5-Dimethylbutylbenzene | MAOA |
| hawthorn | TCMID:6328 | 3,5-Dimethylbutylbenzene | MAOB |
| *P. cuspidatum* | TCMID:3308 | (+)-Catechin | MTTP |
| hawthorn | TCMID:7280 | Eriodictyol-7,3-Diglucoside | MTTP |
| hawthorn | TCMID:6853 | Epicatechin | MTTP |
| *P. cuspidatum* | TCMID:25838 | 3,5-Dimethyl-4-Methoxybenzoic Acid | NFKB1 |
| *P. cuspidatum* | TCMID:25838 | 3,5-Dimethyl-4-Methoxybenzoic Acid | NFKB2 |
| *P. cuspidatum* | TCMID:25838 | 3,5-Dimethyl-4-Methoxybenzoic Acid | NFKBIA |
| hawthorn | TCMID:20265 | Stearin | NPR1 |
| hawthorn | TCMID:6329 | Dimethyl Camphorate | NPR1 |
| *P. cuspidatum* | TCMID:23118 | Trans-Resveratrol | NQO2 |
| hawthorn | TCMID:6329 | Dimethyl Camphorate | NR1H4 |
| *P. cuspidatum* | TCMID:3308 | (+)-Catechin | NR1I2 |
| *P. cuspidatum* | TCMID:8094 | Gallicacid | NR1I2 |
| hawthorn | TCMID:6853 | Epicatechin | NR1I2 |
| *P. cuspidatum* | TCMID:31893 | Quillaic Acid | NR3C1 |
| *P. cuspidatum* | TCMID:25027 | Polygalacic Acid | NR3C1 |
| hawthorn | TCMID:21938 | 3,7,11-Trimethyldodeca-1,7,10-Trien-3-Ol-9-One | NR3C1 |
| hawthorn | TCMID:16283 | 12-Oxoarundoin | NR3C1 |
| hawthorn | TCMID:22254 | Ursolicacid | NR3C1 |
| *P. cuspidatum* | TCMID:31893 | Quillaic Acid | NR3C2 |
| hawthorn | TCMID:12888 | Linoleyl Acetate | NR3C2 |
| hawthorn | TCMID:31632 | Methylheptenone | NR3C2 |
| hawthorn | TCMID:16283 | 12-Oxoarundoin | NR3C2 |
| hawthorn | TCMID:22254 | Ursolicacid | NR3C2 |
| hawthorn | TCMID:31632 | Methylheptenone | NT5E |
| hawthorn | TCMID:7280 | Eriodictyol-7,3-Diglucoside | OAT1 |
| *P. cuspidatum* | TCMID:31893 | Quillaic Acid | OPRK1 |
| hawthorn | TCMID:31632 | Methylheptenone | OPRK1 |
| hawthorn | TCMID:16283 | 12-Oxoarundoin | OPRK1 |
| hawthorn | TCMID:22254 | Ursolicacid | OPRK1 |
| hawthorn | TCMID:14267 | 3-Methyl-1,2-Cyclopentanediol | OPRK1 |
| hawthorn | TCMID:31636 | 3-Methylhistidin | PCK1 |
| hawthorn | TCMID:31632 | Methylheptenone | PDE10A |
| hawthorn | TCMID:30462 | 1-Ethyl-4,8-Dimethoxy-Beta-Carboline | PDE10A |
| hawthorn | TCMID:31632 | Methylheptenone | PDE11A |
| hawthorn | TCMID:31632 | Methylheptenone | PDE1A |
| hawthorn | TCMID:31632 | Methylheptenone | PDE1B |
| hawthorn | TCMID:31632 | Methylheptenone | PDE1C |
| hawthorn | TCMID:31632 | Methylheptenone | PDE2A |
| hawthorn | TCMID:31632 | Methylheptenone | PDE3A |
| hawthorn | TCMID:31632 | Methylheptenone | PDE3B |
| hawthorn | TCMID:31632 | Methylheptenone | PDE4A |
| hawthorn | TCMID:31632 | Methylheptenone | PDE4B |
| hawthorn | TCMID:30462 | 1-Ethyl-4,8-Dimethoxy-Beta-Carboline | PDE4B |
| hawthorn | TCMID:31632 | Methylheptenone | PDE4C |
| hawthorn | TCMID:31632 | Methylheptenone | PDE4D |
| hawthorn | TCMID:30462 | 1-Ethyl-4,8-Dimethoxy-Beta-Carboline | PDE4D |
| hawthorn | TCMID:31632 | Methylheptenone | PDE5A |
| hawthorn | TCMID:31632 | Methylheptenone | PDE6A |
| hawthorn | TCMID:31632 | Methylheptenone | PDE6B |
| hawthorn | TCMID:31632 | Methylheptenone | PDE6C |
| hawthorn | TCMID:31632 | Methylheptenone | PDE7A |
| hawthorn | TCMID:31632 | Methylheptenone | PDE7B |
| hawthorn | TCMID:31632 | Methylheptenone | PDE8A |
| hawthorn | TCMID:31632 | Methylheptenone | PDE8B |
| hawthorn | TCMID:31632 | Methylheptenone | PDE9A |
| hawthorn | TCMID:31632 | Methylheptenone | PGD |
| *P. cuspidatum* | TCMID:31893 | Quillaic Acid | PGR |
| *P. cuspidatum* | TCMID:25027 | Polygalacic Acid | PGR |
| hawthorn | TCMID:12888 | Linoleyl Acetate | PGR |
| hawthorn | TCMID:31632 | Methylheptenone | PGR |
| hawthorn | TCMID:16283 | 12-Oxoarundoin | PGR |
| hawthorn | TCMID:22254 | Ursolicacid | PGR |
| *P. cuspidatum* | TCMID:23118 | Trans-Resveratrol | PHKG2 |
| *P. cuspidatum* | TCMID:18628 | Resveratrol | PHKG2 |
| hawthorn | TCMID:31632 | Methylheptenone | PIK3CA |
| hawthorn | TCMID:31632 | Methylheptenone | PIK3CB |
| hawthorn | TCMID:31632 | Methylheptenone | PIK3CD |
| hawthorn | TCMID:31632 | Methylheptenone | PIK3R1 |
| hawthorn | TCMID:6329 | Dimethyl Camphorate | PLA2G1B |
| hawthorn | TCMID:31632 | Methylheptenone | POLA2 |
| *P. cuspidatum* | TCMID:25838 | 3,5-Dimethyl-4-Methoxybenzoic Acid | PPARA |
| hawthorn | TCMID:21938 | 3,7,11-Trimethyldodeca-1,7,10-Trien-3-Ol-9-One | PPARD |
| hawthorn | TCMID:21938 | 3,7,11-Trimethyldodeca-1,7,10-Trien-3-Ol-9-One | PPARG |
| *P. cuspidatum* | TCMID:3308 | (+)-Catechin | PPP2CA |
| *P. cuspidatum* | TCMID:8094 | Gallicacid | PPP2CA |
| hawthorn | TCMID:6853 | Epicatechin | PPP2CA |
| *P. cuspidatum* | TCMID:3308 | (+)-Catechin | PPP2CB |
| *P. cuspidatum* | TCMID:8094 | Gallicacid | PPP2CB |
| hawthorn | TCMID:6853 | Epicatechin | PPP2CB |
| hawthorn | TCMID:31636 | 3-Methylhistidin | PRG3 |
| *P. cuspidatum* | TCMID:25838 | 3,5-Dimethyl-4-Methoxybenzoic Acid | PRKAA1 |
| *P. cuspidatum* | TCMID:25838 | 3,5-Dimethyl-4-Methoxybenzoic Acid | PRKAA2 |
| *P. cuspidatum* | TCMID:25838 | 3,5-Dimethyl-4-Methoxybenzoic Acid | PRKAB1 |
| hawthorn | TCMID:21949 | 3,4,4-Trimethyl-2-Hexene | PRKAB1 |
| hawthorn | TCMID:14480 | 3-Methylhexane | PRKAB1 |
| hawthorn | TCMID:6317 | 1,2-Dimethylbenzene | PRKAB1 |
| hawthorn | TCMID:14263 | Methylcyclohexane | PRKAB1 |
| hawthorn | TCMID:25839 | 4-(1,5-Dimethyl-1,4-Hexadienyl)-1-Methyl-Cyclohexene | PRKAB1 |
| *P. cuspidatum* | TCMID:25838 | 3,5-Dimethyl-4-Methoxybenzoic Acid | PRKAB2 |
| *P. cuspidatum* | TCMID:25838 | 3,5-Dimethyl-4-Methoxybenzoic Acid | PRKAG1 |
| *P. cuspidatum* | TCMID:25838 | 3,5-Dimethyl-4-Methoxybenzoic Acid | PRKAG2 |
| *P. cuspidatum* | TCMID:25838 | 3,5-Dimethyl-4-Methoxybenzoic Acid | PRKAG3 |
| *P. cuspidatum* | TCMID:3308 | (+)-Catechin | PRKCA |
| *P. cuspidatum* | TCMID:8094 | Gallicacid | PRKCA |
| hawthorn | TCMID:9488 | 20-Hexadecanoylingenol | PRKCA |
| hawthorn | TCMID:6853 | Epicatechin | PRKCA |
| *P. cuspidatum* | TCMID:3308 | (+)-Catechin | PRKCB |
| *P. cuspidatum* | TCMID:8094 | Gallicacid | PRKCB |
| hawthorn | TCMID:6853 | Epicatechin | PRKCB |
| hawthorn | TCMID:9488 | 20-Hexadecanoylingenol | PRKCD |
| hawthorn | TCMID:31632 | Methylheptenone | PRKDC |
| *P. cuspidatum* | TCMID:25027 | Polygalacic Acid | PRLR |
| hawthorn | TCMID:16283 | 12-Oxoarundoin | PRLR |
| hawthorn | TCMID:22254 | Ursolicacid | PRLR |
| *P. cuspidatum* | TCMID:31893 | Quillaic Acid | PTGER1 |
| *P. cuspidatum* | TCMID:25027 | Polygalacic Acid | PTGER1 |
| hawthorn | TCMID:22254 | Ursolicacid | PTGER1 |
| *P. cuspidatum* | TCMID:31893 | Quillaic Acid | PTGER2 |
| *P. cuspidatum* | TCMID:25027 | Polygalacic Acid | PTGER2 |
| hawthorn | TCMID:16283 | 12-Oxoarundoin | PTGER2 |
| hawthorn | TCMID:22254 | Ursolicacid | PTGER2 |
| *P. cuspidatum* | TCMID:31893 | Quillaic Acid | PTGER3 |
| *P. cuspidatum* | TCMID:25027 | Polygalacic Acid | PTGER3 |
| hawthorn | TCMID:16283 | 12-Oxoarundoin | PTGER3 |
| hawthorn | TCMID:22254 | Ursolicacid | PTGER3 |
| *P. cuspidatum* | TCMID:31893 | Quillaic Acid | PTGER4 |
| *P. cuspidatum* | TCMID:25027 | Polygalacic Acid | PTGER4 |
| hawthorn | TCMID:16283 | 12-Oxoarundoin | PTGER4 |
| hawthorn | TCMID:22254 | Ursolicacid | PTGER4 |
| *P. cuspidatum* | TCMID:1367 | Anthraquinone | PTGIS |
| *P. cuspidatum* | TCMID:31893 | Quillaic Acid | PTGS1 |
| *P. cuspidatum* | TCMID:25838 | 3,5-Dimethyl-4-Methoxybenzoic Acid | PTGS1 |
| *P. cuspidatum* | TCMID:23118 | Trans-Resveratrol | PTGS1 |
| *P. cuspidatum* | TCMID:25027 | Polygalacic Acid | PTGS1 |
| *P. cuspidatum* | TCMID:8094 | Gallicacid | PTGS1 |
| *P. cuspidatum* | TCMID:1367 | Anthraquinone | PTGS1 |
| hawthorn | TCMID:12888 | Linoleyl Acetate | PTGS1 |
| hawthorn | TCMID:21938 | 3,7,11-Trimethyldodeca-1,7,10-Trien-3-Ol-9-One | PTGS1 |
| hawthorn | TCMID:22254 | Ursolicacid | PTGS1 |
| *P. cuspidatum* | TCMID:31893 | Quillaic Acid | PTGS2 |
| *P. cuspidatum* | TCMID:25838 | 3,5-Dimethyl-4-Methoxybenzoic Acid | PTGS2 |
| *P. cuspidatum* | TCMID:23118 | Trans-Resveratrol | PTGS2 |
| *P. cuspidatum* | TCMID:25027 | Polygalacic Acid | PTGS2 |
| *P. cuspidatum* | TCMID:8094 | Gallicacid | PTGS2 |
| *P. cuspidatum* | TCMID:1367 | Anthraquinone | PTGS2 |
| hawthorn | TCMID:12888 | Linoleyl Acetate | PTGS2 |
| hawthorn | TCMID:21938 | 3,7,11-Trimethyldodeca-1,7,10-Trien-3-Ol-9-One | PTGS2 |
| hawthorn | TCMID:22254 | Ursolicacid | PTGS2 |
| *P. cuspidatum* | TCMID:25838 | 3,5-Dimethyl-4-Methoxybenzoic Acid | RARA |
| hawthorn | TCMID:23381 | Caffeic Acid Dimethyl Ether | RARA |
| *P. cuspidatum* | TCMID:25838 | 3,5-Dimethyl-4-Methoxybenzoic Acid | RARB |
| hawthorn | TCMID:23381 | Caffeic Acid Dimethyl Ether | RARB |
| *P. cuspidatum* | TCMID:25838 | 3,5-Dimethyl-4-Methoxybenzoic Acid | RARG |
| hawthorn | TCMID:23381 | Caffeic Acid Dimethyl Ether | RARG |
| hawthorn | TCMID:23381 | Caffeic Acid Dimethyl Ether | RBP1 |
| hawthorn | TCMID:31632 | Methylheptenone | RINT1 |
| hawthorn | TCMID:31632 | Methylheptenone | RIPK1 |
| hawthorn | TCMID:21949 | 3,4,4-Trimethyl-2-Hexene | RNASE1 |
| hawthorn | TCMID:14480 | 3-Methylhexane | RNASE1 |
| hawthorn | TCMID:6317 | 1,2-Dimethylbenzene | RNASE1 |
| hawthorn | TCMID:14263 | Methylcyclohexane | RNASE1 |
| hawthorn | TCMID:25839 | 4-(1,5-Dimethyl-1,4-Hexadienyl)-1-Methyl-Cyclohexene | RNASE1 |
| *P. cuspidatum* | TCMID:25838 | 3,5-Dimethyl-4-Methoxybenzoic Acid | RPS6KA3 |
| *P. cuspidatum* | TCMID:25838 | 3,5-Dimethyl-4-Methoxybenzoic Acid | RXRA |
| hawthorn | TCMID:23381 | Caffeic Acid Dimethyl Ether | RXRA |
| *P. cuspidatum* | TCMID:25838 | 3,5-Dimethyl-4-Methoxybenzoic Acid | RXRB |
| hawthorn | TCMID:23381 | Caffeic Acid Dimethyl Ether | RXRB |
| *P. cuspidatum* | TCMID:25838 | 3,5-Dimethyl-4-Methoxybenzoic Acid | RXRG |
| hawthorn | TCMID:23381 | Caffeic Acid Dimethyl Ether | RXRG |
| hawthorn | TCMID:31632 | Methylheptenone | RYR1 |
| hawthorn | TCMID:6328 | 3,5-Dimethylbutylbenzene | S1PR5 |
| *P. cuspidatum* | TCMID:3308 | (+)-Catechin | SEC14L2 |
| *P. cuspidatum* | TCMID:8094 | Gallicacid | SEC14L2 |
| hawthorn | TCMID:6853 | Epicatechin | SEC14L2 |
| *P. cuspidatum* | TCMID:3308 | (+)-Catechin | SEC14L3 |
| *P. cuspidatum* | TCMID:8094 | Gallicacid | SEC14L3 |
| hawthorn | TCMID:6853 | Epicatechin | SEC14L3 |
| *P. cuspidatum* | TCMID:3308 | (+)-Catechin | SEC14L4 |
| *P. cuspidatum* | TCMID:8094 | Gallicacid | SEC14L4 |
| hawthorn | TCMID:6853 | Epicatechin | SEC14L4 |
| *P. cuspidatum* | TCMID:25838 | 3,5-Dimethyl-4-Methoxybenzoic Acid | SERPINB7 |
| *P. cuspidatum* | TCMID:1367 | Anthraquinone | SERPINB7 |
| *P. cuspidatum* | TCMID:23118 | Trans-Resveratrol | SIRT1 |
| *P. cuspidatum* | TCMID:7713 | Fangchinoline | SLC18A2 |
| hawthorn | TCMID:6328 | 3,5-Dimethylbutylbenzene | SLC18A2 |
| hawthorn | TCMID:31636 | 3-Methylhistidin | SLC38A3 |
| hawthorn | TCMID:31636 | 3-Methylhistidin | SLC38A7 |
| hawthorn | TCMID:6328 | 3,5-Dimethylbutylbenzene | SLC6A2 |
| hawthorn | TCMID:6328 | 3,5-Dimethylbutylbenzene | SLC6A3 |
| hawthorn | TCMID:6328 | 3,5-Dimethylbutylbenzene | SLC6A4 |
| *P. cuspidatum* | TCMID:31893 | Quillaic Acid | SLC8A1 |
| *P. cuspidatum* | TCMID:25027 | Polygalacic Acid | SLC8A1 |
| hawthorn | TCMID:12888 | Linoleyl Acetate | SLC8A1 |
| hawthorn | TCMID:21938 | 3,7,11-Trimethyldodeca-1,7,10-Trien-3-Ol-9-One | SLC8A1 |
| hawthorn | TCMID:22254 | Ursolicacid | SLC8A1 |
| *P. cuspidatum* | TCMID:3308 | (+)-Catechin | SOAT |
| hawthorn | TCMID:6853 | Epicatechin | SOAT1 |
| *P. cuspidatum* | TCMID:3308 | (+)-Catechin | SOAT2 |
| hawthorn | TCMID:7280 | Eriodictyol-7,3-Diglucoside | SOAT2 |
| hawthorn | TCMID:6853 | Epicatechin | SOAT2 |
| *P. cuspidatum* | TCMID:31893 | Quillaic Acid | SRD5A1 |
| *P. cuspidatum* | TCMID:25027 | Polygalacic Acid | SRD5A1 |
| hawthorn | TCMID:16283 | 12-Oxoarundoin | SRD5A1 |
| hawthorn | TCMID:22254 | Ursolicacid | SRD5A1 |
| hawthorn | TCMID:6329 | Dimethyl Camphorate | SRD5A2 |
| hawthorn | TCMID:6328 | 3,5-Dimethylbutylbenzene | TAAR1 |
| hawthorn | TCMID:31632 | Methylheptenone | TACR2 |
| *P. cuspidatum* | TCMID:3615 | Chrysophanol | TCAF1 |
| *P. cuspidatum* | TCMID:25838 | 3,5-Dimethyl-4-Methoxybenzoic Acid | TNF |
| hawthorn | TCMID:23381 | Caffeic Acid Dimethyl Ether | TNF |
| *P. cuspidatum* | TCMID:25838 | 3,5-Dimethyl-4-Methoxybenzoic Acid | TP53 |
| hawthorn | TCMID:21949 | 3,4,4-Trimethyl-2-Hexene | TPO |
| hawthorn | TCMID:14480 | 3-Methylhexane | TPO |
| hawthorn | TCMID:6317 | 1,2-Dimethylbenzene | TPO |
| hawthorn | TCMID:14263 | Methylcyclohexane | TPO |
| hawthorn | TCMID:25839 | 4-(1,5-Dimethyl-1,4-Hexadienyl)-1-Methyl-Cyclohexene | TPO |
| hawthorn | TCMID:14267 | 3-Methyl-1,2-Cyclopentanediol | TRPA1 |
| hawthorn | TCMID:14267 | 3-Methyl-1,2-Cyclopentanediol | TRPM8 |
| *P. cuspidatum* | TCMID:31893 | Quillaic Acid | TRPV1 |
| *P. cuspidatum* | TCMID:25027 | Polygalacic Acid | TRPV1 |
| hawthorn | TCMID:12888 | Linoleyl Acetate | TRPV1 |
| hawthorn | TCMID:21938 | 3,7,11-Trimethyldodeca-1,7,10-Trien-3-Ol-9-One | TRPV1 |
| hawthorn | TCMID:22254 | Ursolicacid | TRPV1 |
| hawthorn | TCMID:14267 | 3-Methyl-1,2-Cyclopentanediol | TRPV3 |
| *P. cuspidatum* | TCMID:23118 | Trans-Resveratrol | TYR |
| *P. cuspidatum* | TCMID:18628 | Resveratrol | TYR |
| hawthorn | TCMID:6329 | Dimethyl Camphorate | TYR |
| *P. cuspidatum* | TCMID:1367 | Anthraquinone | VKORC1 |
| *P. cuspidatum* | TCMID:1367 | Anthraquinone | VKORC1L1 |
| hawthorn | TCMID:31636 | 3-Methylhistidin | WARS |
| hawthorn | TCMID:31636 | 3-Methylhistidin | WARS2 |
| hawthorn | TCMID:31632 | Methylheptenone | WNT4 |

**Supplementary Table S5:** Results of ligand-receptor protein molecular docking (a-j).

| mode | affinity(kcal/mol) | dist from best mode | |
| --- | --- | --- | --- |
|  |  | rmsd l.b. | rmsd u.b. |
| 1 | -7.8 | 0 | 0 |
| 2 | -7.8 | 0.106 | 2.362 |
| 3 | -6.7 | 3.968 | 6.232 |
| 4 | -6.7 | 1.756 | 4.432 |
| 5 | -6.7 | 3.953 | 6.247 |
| 6 | -6.7 | 3.364 | 3.955 |
| 7 | -6.6 | 1.594 | 5.144 |
| 8 | -6.6 | 3.479 | 4.074 |
| 9 | -6.6 | 2.334 | 3.847 |
| 10 | -6.5 | 19.358 | 20.768 |
| 11 | -6.5 | 19.56 | 21.109 |
| 12 | -6.1 | 3.321 | 5.125 |
| 13 | -6 | 2.445 | 3.401 |
| 14 | -6 | 5.4 | 7.389 |
| 15 | -6 | 20.585 | 22.145 |
| 16 | -5.8 | 15.469 | 17.245 |
| 17 | -5.8 | 3.741 | 6.042 |
| 18 | -5.8 | 3.699 | 5.637 |
| 19 | -5.7 | 4.383 | 8.164 |
| 20 | -5.7 | 15.474 | 17.309 |

8a: ligand-receptor protein molecular docking score between TNF and 3,5-Dimethyl-4-Methoxybenzoic Acid

| mode | affinity(kcal/mol) | dist from best mode | |
| --- | --- | --- | --- |
|  |  | rmsd l.b. | rmsd u.b. |
| 1 | -7.6 | 0 | 0 |
| 2 | -7.2 | 1.693 | 5.877 |
| 3 | -6.9 | 3.068 | 6.394 |
| 4 | -6.8 | 2.714 | 5.185 |
| 5 | -6.4 | 3.191 | 6.149 |
| 6 | -6.3 | 1.852 | 6.192 |
| 7 | -6.2 | 14.39 | 15.618 |
| 8 | -6.1 | 12.772 | 14.849 |
| 9 | -6.1 | 2.527 | 5.586 |
| 10 | -6 | 3.621 | 6.569 |
| 11 | -6 | 2.087 | 6.29 |
| 12 | -6 | 4.216 | 4.844 |
| 13 | -6 | 2.797 | 4.472 |
| 14 | -5.7 | 2.384 | 6.155 |
| 15 | -5.7 | 2.594 | 6.759 |
| 16 | -5.7 | 2.337 | 6.452 |
| 17 | -5.7 | 15.298 | 17.882 |
| 18 | -5.6 | 2.967 | 6.807 |
| 19 | -5.6 | 15.096 | 17.933 |
| 20 | -5.6 | 2.723 | 3.554 |

8b: ligand-receptor protein molecular docking score between TNF and Caffeic Acid Dimethyl Ether

| mode | affinity(kcal/mol) | dist from best mode | |
| --- | --- | --- | --- |
|  |  | rmsd l.b. | rmsd u.b. |
| 1 | -4.5 | 0 | 0 |
| 2 | -4.5 | 0.299 | 2.376 |
| 3 | -4.4 | 2.968 | 4.957 |
| 4 | -4.2 | 1.816 | 4.083 |
| 5 | -4.2 | 23.94 | 25.552 |
| 6 | -4.2 | 27.025 | 27.842 |
| 7 | -4.1 | 26.994 | 27.864 |
| 8 | -4.1 | 19.838 | 20.62 |
| 9 | -4.1 | 14.088 | 16.217 |
| 10 | -4.1 | 23.968 | 25.652 |
| 11 | -4.1 | 27.54 | 28.564 |
| 12 | -4.1 | 2.685 | 4.085 |
| 13 | -4.1 | 25.476 | 26.837 |
| 14 | -4.1 | 25.536 | 26.726 |
| 15 | -4.1 | 1.53 | 4.618 |
| 16 | -4 | 2.757 | 4.697 |
| 17 | -4 | 14.436 | 16.576 |
| 18 | -4 | 27.416 | 28.502 |
| 19 | -3.9 | 2.38 | 4.926 |
| 20 | -3.9 | 13.594 | 15.744 |

8c: ligand-receptor protein molecular docking score between NF-κB1 and 3,5-Dimethyl-4-Methoxybenzoic Acid

| mode | affinity(kcal/mol) | dist from best mode | |
| --- | --- | --- | --- |
|  |  | rmsd l.b. | rmsd u.b. |
| 1 | -6.4 | 0 | 0 |
| 2 | -6.3 | 2.323 | 7.927 |
| 3 | -6.3 | 2.596 | 5.577 |
| 4 | -6.1 | 25.765 | 28.298 |
| 5 | -6 | 24.887 | 28.334 |
| 6 | -5.8 | 2.211 | 4.476 |
| 7 | -5.8 | 25.45 | 29.218 |
| 8 | -5.7 | 25.41 | 27.69 |
| 9 | -5.7 | 25.501 | 28.408 |
| 10 | -5.7 | 21.493 | 25.387 |
| 11 | -5.6 | 2.517 | 7.64 |
| 12 | -5.6 | 25.94 | 29.279 |
| 13 | -5.5 | 2.644 | 5.418 |
| 14 | -5.5 | 3.389 | 5.508 |
| 15 | -5.5 | 25.762 | 28.71 |
| 16 | -5.5 | 25.621 | 28.643 |
| 17 | -5.5 | 23.857 | 27.379 |
| 18 | -5.4 | 23.474 | 26.592 |
| 19 | -5.4 | 26.541 | 29.913 |
| 20 | -5.3 | 24.742 | 28.323 |

8d: ligand-receptor protein molecular docking score between NF-κB1 and Simvastatin

| mode | affinity(kcal/mol) | dist from best mode | |
| --- | --- | --- | --- |
|  |  | rmsd l.b. | rmsd u.b. |
| 1 | -8.2 | 0 | 0 |
| 2 | -8.2 | 36.021 | 39.944 |
| 3 | -8 | 37.526 | 41.263 |
| 4 | -7.9 | 34.944 | 36.588 |
| 5 | -7.9 | 1.97 | 5.022 |
| 6 | -7.8 | 34.715 | 36.772 |
| 7 | -7.8 | 1.825 | 3.393 |
| 8 | -7.6 | 1.454 | 6.056 |
| 9 | -7.4 | 32.513 | 36.177 |
| 10 | -7.1 | 32.195 | 34.246 |
| 11 | -7.1 | 32.236 | 34.474 |
| 12 | -6.9 | 32.102 | 34.427 |
| 13 | -6.9 | 23.517 | 26.461 |
| 14 | -6.9 | 20.793 | 24.301 |
| 15 | -6.8 | 34.679 | 38.129 |
| 16 | -6.7 | 31.388 | 34.005 |
| 17 | -6.5 | 20.622 | 22.346 |
| 18 | -6.4 | 21.365 | 24.412 |
| 19 | -6.2 | 31.182 | 33.482 |
| 1 | -8.2 | 0 | 0 |

8e: ligand-receptor protein molecular docking score between ESR1 and Emodin

| mode | affinity(kcal/mol) | dist from best mode | |
| --- | --- | --- | --- |
|  |  | rmsd l.b. | rmsd u.b. |
| 1 | -7.4 | 0 | 0 |
| 2 | -7.3 | 14.626 | 18.373 |
| 3 | -7.1 | 5.368 | 8.077 |
| 4 | -7 | 10.098 | 12.887 |
| 5 | -6.9 | 6.345 | 8.411 |
| 6 | -6.9 | 9.35 | 13.18 |
| 7 | -6.8 | 9.791 | 12.346 |
| 8 | -6.7 | 17.648 | 21.969 |
| 9 | -6.7 | 8.78 | 12.437 |
| 10 | -6.5 | 18.82 | 21.36 |
| 11 | -6.5 | 4.469 | 6.548 |
| 12 | -6.5 | 14.01 | 16.484 |
| 13 | -6.5 | 9.928 | 13.476 |
| 14 | -6.4 | 4.876 | 8.183 |
| 15 | -6.3 | 5.138 | 9.193 |
| 16 | -6.2 | 11.051 | 14.016 |
| 17 | -6.1 | 8.055 | 11.974 |
| 18 | -6.1 | 3.883 | 7.598 |
| 19 | -6.1 | 7.77 | 10.331 |
| 20 | -5.9 | 15.057 | 18.027 |

8f: ligand-receptor protein molecular docking score between ESR1 and Polygalacic Acid

| mode | affinity(kcal/mol) | dist from best mode | |
| --- | --- | --- | --- |
|  |  | rmsd l.b. | rmsd u.b. |
| 1 | -7.9 | 0 | 0 |
| 2 | -7.8 | 12.71 | 15.098 |
| 3 | -7.8 | 15.2 | 18.613 |
| 4 | -7.6 | 4.954 | 10.245 |
| 5 | -7.4 | 3.292 | 8.146 |
| 6 | -7.3 | 17.48 | 21.18 |
| 7 | -7.3 | 14.715 | 16.486 |
| 8 | -7.1 | 12.864 | 14.95 |
| 9 | -7 | 8.558 | 12.746 |
| 10 | -7 | 9.543 | 13.45 |
| 11 | -6.9 | 6.23 | 8.753 |
| 12 | -6.9 | 12.778 | 15.24 |
| 13 | -6.8 | 6.446 | 9.962 |
| 14 | -6.7 | 7.174 | 9.579 |
| 15 | -6.7 | 7.926 | 9.84 |
| 16 | -6.7 | 4.34 | 7.266 |
| 17 | -6.6 | 7.239 | 11.898 |
| 18 | -6.6 | 16.929 | 19.868 |
| 19 | -6.6 | 9.375 | 12.6 |
| 20 | -6.5 | 8.576 | 10.56 |

8g: ligand-receptor protein molecular docking score between ESR1 and Quillaic Acid

| mode | affinity(kcal/mol) | dist from best mode | |
| --- | --- | --- | --- |
|  |  | rmsd l.b. | rmsd u.b. |
| 1 | -7.2 | 0 | 0 |
| 2 | -7.1 | 11.645 | 12.773 |
| 3 | -7 | 1.205 | 7.2 |
| 4 | -7 | 11.168 | 12.558 |
| 5 | -7 | 1.222 | 7.387 |
| 6 | -7 | 11.235 | 12.496 |
| 7 | -7 | 11.012 | 13.511 |
| 8 | -6.9 | 1.092 | 2.336 |
| 9 | -6.8 | 1.233 | 7.23 |
| 10 | -6.7 | 11.659 | 13.358 |
| 11 | -6.7 | 11.548 | 12.312 |
| 12 | -6.7 | 1.154 | 2.156 |
| 13 | -6.4 | 11.083 | 12.356 |
| 14 | -6.1 | 10.471 | 11.708 |
| 15 | -6.1 | 22.462 | 26.421 |
| 16 | -6 | 22.59 | 26.778 |
| 17 | -5.7 | 10.869 | 12.414 |
| 18 | -5.6 | 11.345 | 12.344 |
| 19 | -5.4 | 12.498 | 15.708 |
| 20 | -5.3 | 9.838 | 13.303 |

8h: ligand-receptor protein molecular docking score between ESR1 and Resveratrol

| mode | affinity(kcal/mol) | dist from best mode | |
| --- | --- | --- | --- |
|  |  | rmsd l.b. | rmsd u.b. |
| 1 | -9.3 | 0 | 0 |
| 2 | -7.7 | 24.434 | 27.55 |
| 3 | -7.6 | 16.003 | 19.491 |
| 4 | -7.5 | 27.018 | 30.987 |
| 5 | -7.5 | 17.339 | 20.813 |
| 6 | -7.5 | 15.37 | 18.83 |
| 7 | -7.5 | 29.442 | 32.321 |
| 8 | -7.4 | 28.192 | 32 |
| 9 | -7.4 | 29.205 | 32.6 |
| 10 | -7.3 | 18.391 | 20.975 |
| 11 | -7.3 | 29.096 | 32.399 |
| 12 | -7.3 | 28.868 | 32.005 |
| 13 | -7.2 | 16.265 | 19.986 |
| 14 | -7.2 | 26.967 | 30.245 |
| 15 | -7.2 | 16.78 | 19.952 |
| 16 | -7 | 19.526 | 22.756 |
| 17 | -7 | 27.58 | 30.724 |
| 18 | -7 | 29.958 | 33.155 |
| 19 | -6.9 | 28.515 | 31.585 |
| 20 | -6.8 | 24.343 | 27.652 |

8i: ligand-receptor protein molecular docking score between ESR1 and Simvastatin

| mode | affinity(kcal/mol) | dist from best mode | |
| --- | --- | --- | --- |
|  |  | rmsd l.b. | rmsd u.b. |
| 1 | -7.9 | 0 | 0 |
| 2 | -7.6 | 13.142 | 15.28 |
| 3 | -7.5 | 7 | 10.356 |
| 4 | -7.5 | 10.914 | 13.346 |
| 5 | -7.5 | 6.218 | 11.023 |
| 6 | -7.4 | 7.048 | 10.689 |
| 7 | -7.2 | 12.826 | 15.646 |
| 8 | -7.1 | 10.726 | 12.931 |
| 9 | -6.8 | 12.189 | 15.23 |
| 10 | -6.7 | 13.456 | 16.909 |
| 11 | -6.7 | 11.528 | 14.496 |
| 12 | -6.7 | 2.285 | 8.005 |
| 13 | -6.6 | 11.332 | 14.492 |
| 14 | -6.5 | 9.637 | 12.016 |
| 15 | -6.4 | 16.072 | 19.716 |
| 16 | -6.4 | 10.412 | 13.685 |
| 17 | -6.4 | 9.447 | 12.21 |
| 18 | -6.3 | 2.021 | 7.55 |
| 19 | -6.3 | 12.616 | 16 |
| 20 | -6.3 | 11.889 | 15.451 |

8j: ligand-receptor protein molecular docking score between ESR1 and Ursolicacid

**Supplementary Table S6:** signaling pathway in CHD.


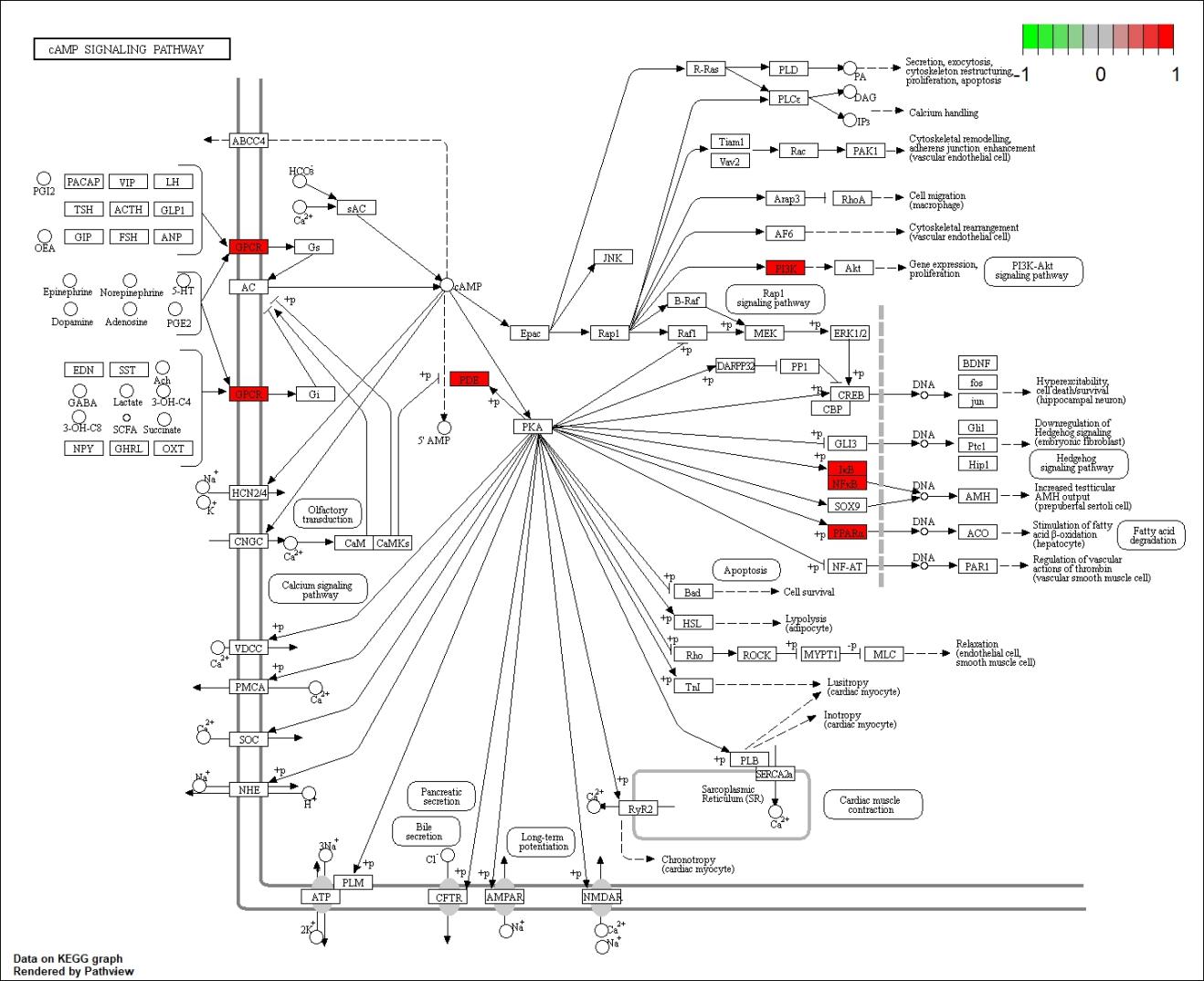


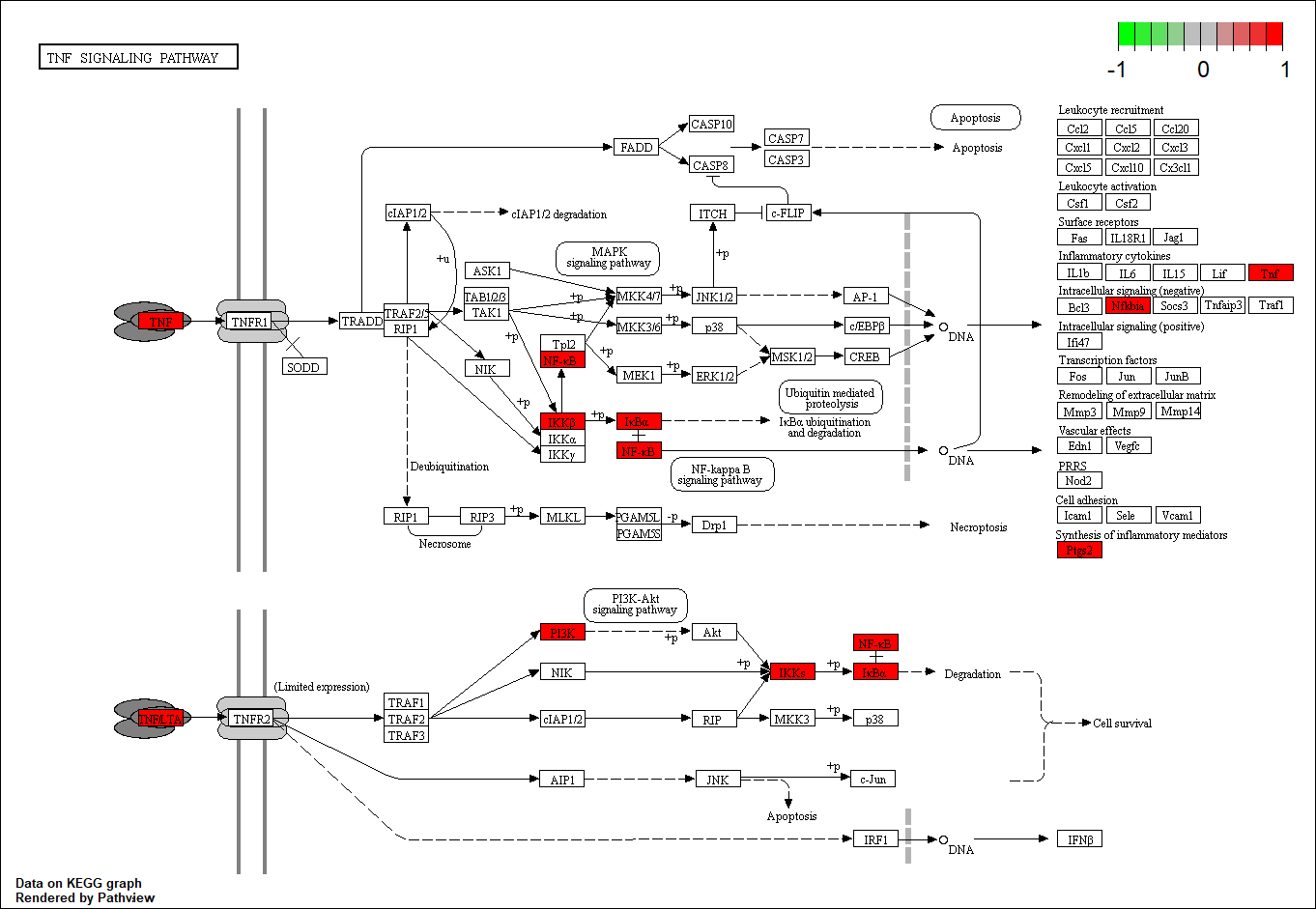


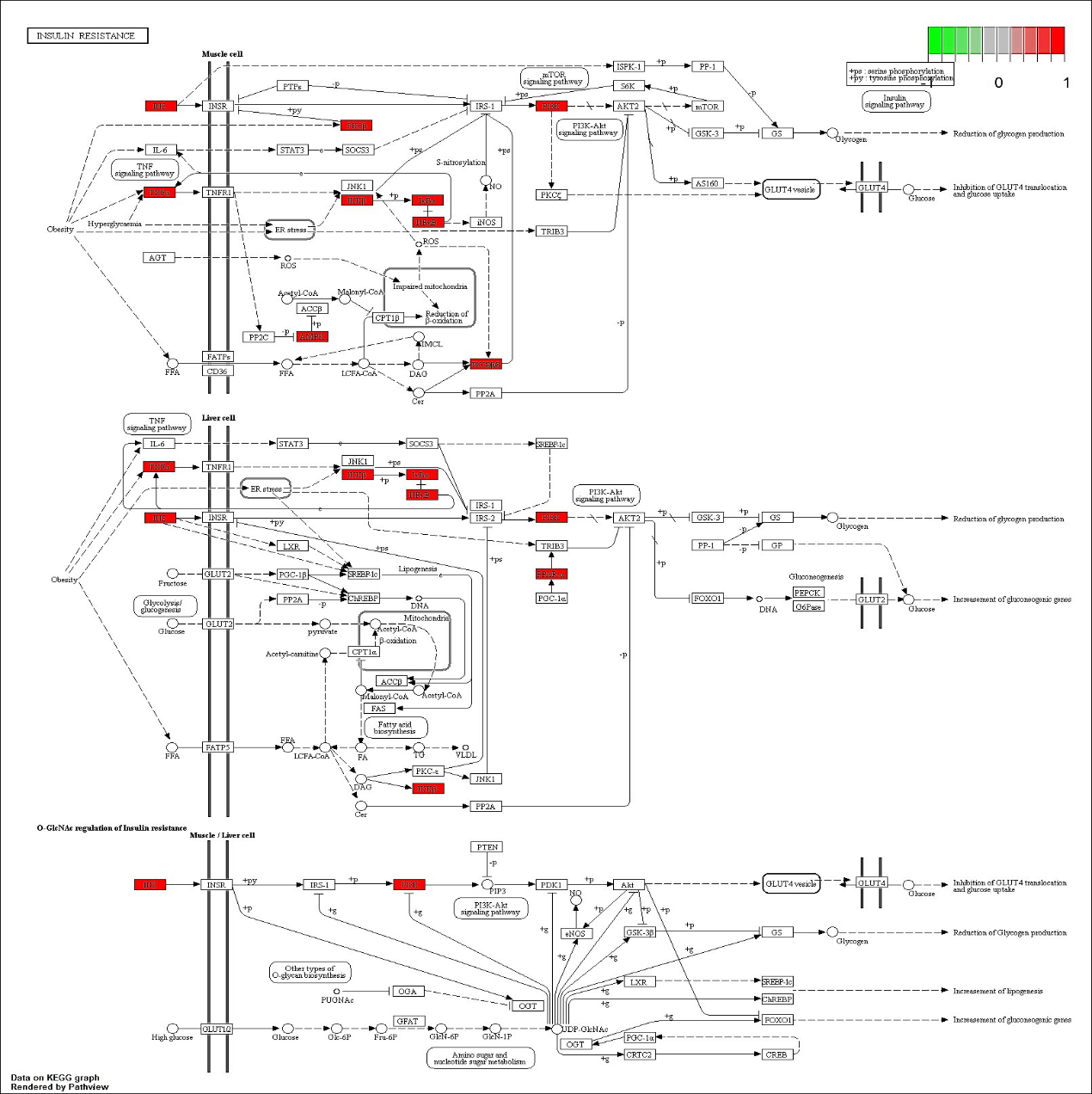


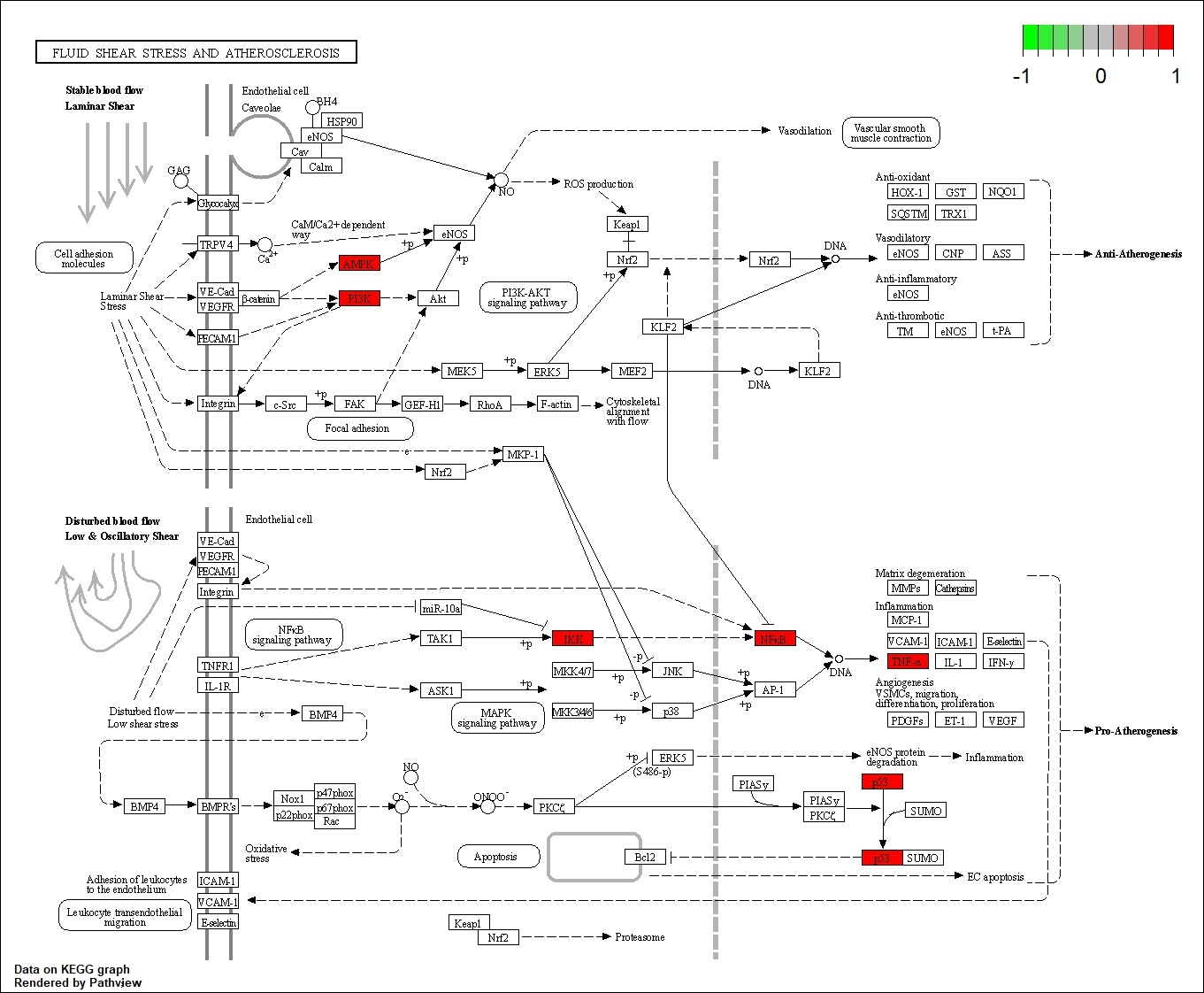


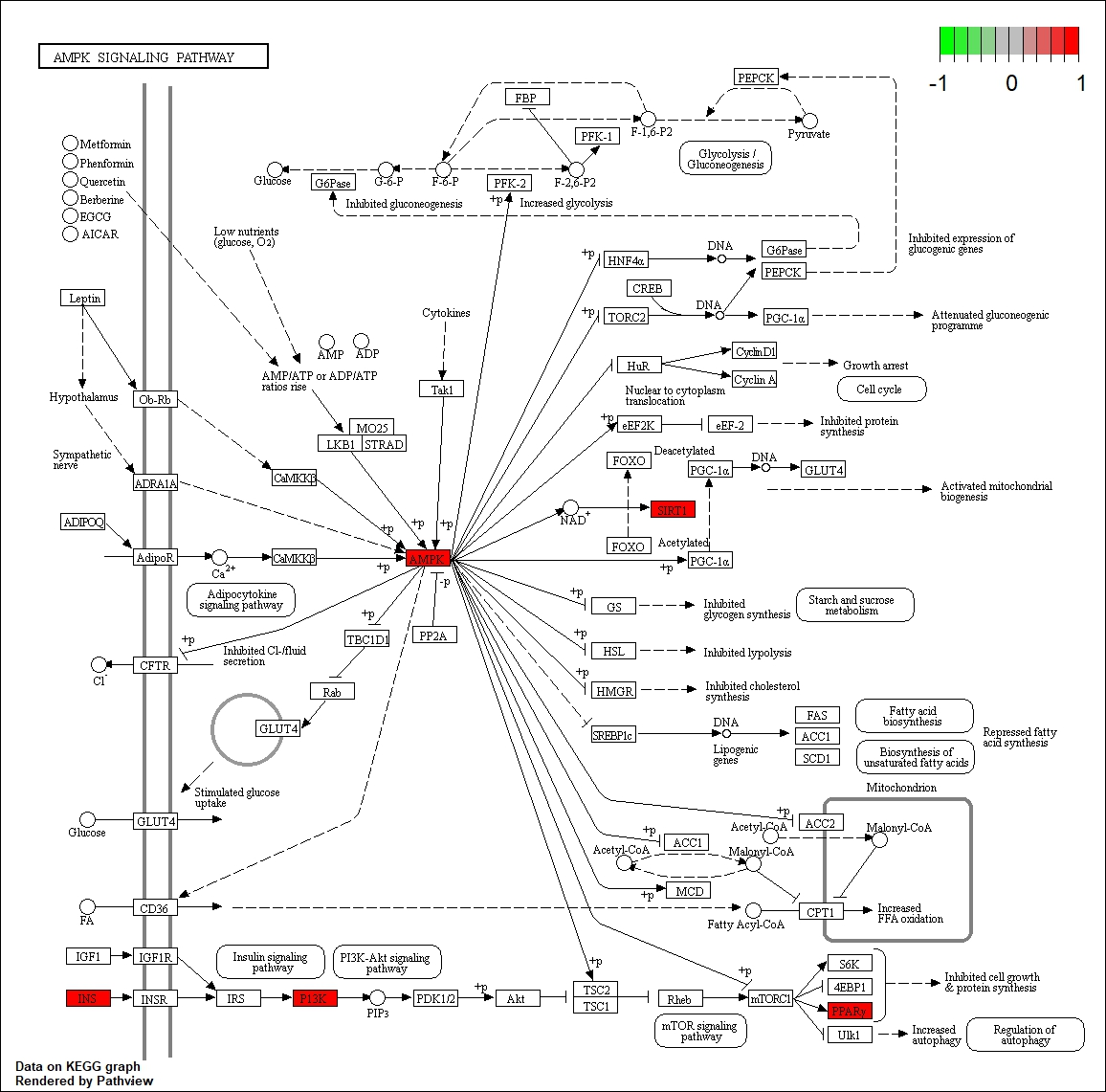


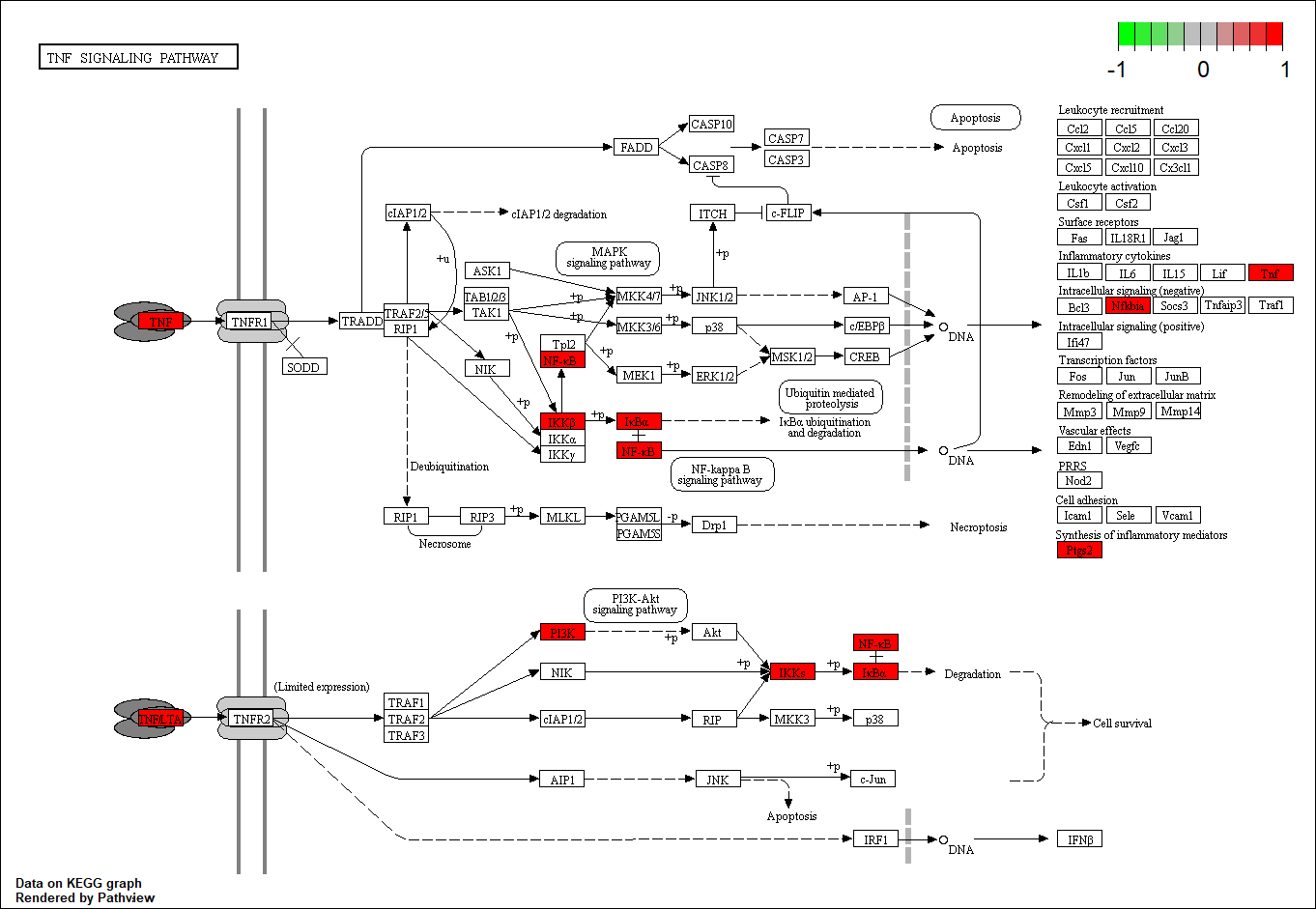

Supplement: Supplementary Materials — Supplementary Table S1: The information of all ingredients of HSHP for coronary heart disease. Supplementary Table S2: Active ingredient parameters of HSHP. Supplementary Table S3: All genes of HSHP in the treatment of CHD. Supplementary Table S4: Compound-target pair information. Supplementary Table S5: Results of ligand-receptor protein molecular docking. Supplementary Table S6: signalling pathway in CHD. [file 5569666.f1.docx]
